# CYP450 Constraints in Infants: A Multidomain Convergence Framework for Investigating Mechanistic Vulnerability

**DOI:** 10.7150/ijms.129127

**Published:** 2026-02-26

**Authors:** Gary S. Goldman

**Affiliations:** Independent Researcher, 1882 Mill Creek Ln SW, Bogue Chitto, MS 39629.

**Keywords:** CYP450, metabolic vulnerability, phenoconversion, cytokines, redox balance, sudden unexpected infant death (SUID), metabolic vulnerability index

## Abstract

Aim and background: Cytochrome P450 (CYP450) enzymes are the primary hepatic Phase I oxidative biotransformation system for many drugs and xenobiotics; variability in CYP capacity is therefore a key determinant of metabolic reserve. Reserve varies with developmental ontogeny, genotype, and acquired suppression (e.g., cytokine-mediated phenoconversion). Early infancy represents a developmental window in which clearance capacity and redox/energetic buffering may be comparatively constrained. We introduce a hypothesis-generating Three-Axis Convergence Framework (TCF) modeling interacting effects of (i) developmental/genetic reserve limits, (ii) immune-cytokine modulation of metabolism, and (iii) exposure/disposition context, and translate this synthesis into a structured postmortem interpretive tool integrated into the primary medicolegal autopsy, using an enhanced analytic panel applied selectively based on case context and specimen validity/QC to support mechanistic characterization of infant deaths remaining unexplained (often SUID/SIDS).

Methods: A structured narrative synthesis was conducted spanning developmental pharmacology, pharmacogenetics, immunology, redox biology, neuropathology, and toxicology. Routine medicolegal postmortem practices used in SUID investigations were reviewed to identify measurement gaps that may limit mechanistic resolution in unexplained cases. The synthesis was formalized into five analytic domains: CYP450 capacity, immune/cytokine load, redox balance/energetics, neurochemical integrity, and xenobiotic/metal burden.

Results: The Metabolic Vulnerability Index (MVI) operationalizes the TCF as a five-domain ordinal scoring system (0-15). Domain 1 is anchored by hepatic CYP protein abundance (a more postmortem-stable proxy than CYP activity assays, which are generally constrained by rapid functional decay and QC limitations), normalized to adult reference and interpreted against age-matched developmental expectations. Domain-combination lookup tables route users to 14 mechanistically defined archetypes and specify modifier/exposure-context documentation. Appendices define an operational postmortem workflow, specimen validity rules, analytic QC constraints, detection limits, and a worked example. A Cytokine-Metabolic Suppression Profile (CMSP) is presented as an interpretive coherence summary and does not modify MVI scoring or certification.

Conclusion: The MVI provides a structured framework for describing multidomain physiologic constraints in unexplained infant deaths alongside standard forensic practice. In this way, the TCF, MVI, and CMSP together offer a disciplined response to long-standing mechanistic uncertainty in early life—by enabling systematic measurement, coherent interpretation, and transparent identification of evidence gaps, rather than asserting new causes.

## 1. Introduction

Cytochrome P450 (CYP450) enzymes constitute a major enzymatic system for the oxidative (Phase I) biotransformation of many medications and environmental chemicals, and they also contribute to the metabolism of numerous endogenous substrates, including steroid hormones, fatty acids, bile acids, and select neuroactive compounds such as certain neurosteroids. The CYP450 superfamily comprises 57 functional genes, each encoding a corresponding isoform—such as CYP3A4, CYP2D6, or CYP2C19—with characteristic, though non-exclusive, substrate preferences; CYP-mediated Phase I oxidation is often followed by Phase II conjugation steps (e.g., glucuronidation or sulfation) and transporter-mediated elimination. CYP450 enzymes are expressed predominantly in the endoplasmic reticulum of hepatocytes but also contribute to metabolism in extrahepatic tissues (including intestine, lung, and kidney, and to a more limited extent the central nervous system), with additional activity in select mitochondrial CYP systems, supporting organ-specific patterns of metabolic handling 1-4. In practical terms, CYP450 capacity is a key determinant of metabolic reserve—how rapidly relevant substrates are biotransformed and cleared under varying physiologic conditions. A more detailed overview of CYP450 monooxygenase function and catalytic cycling is provided in Appendix A.

In this review, metabolic vulnerability refers to a developmentally modulated constraint on physiologic reserve in which metabolic demands may transiently outpace functional clearance capacity and redox buffering, thereby narrowing tolerance to additional physiologic stressors. This concept is most salient in early life, when multiple oxidative pathways are relatively immature and intercurrent illness or competing substrates (e.g., medications) can further reduce effective metabolic capacity.

The clinical relevance of CYP450 variability is well established in adolescent and adult psychopharmacology. Inherited variation in CYP2D6 and CYP2C19 can produce substantial differences in plasma concentrations of antidepressants, antipsychotics, stimulants, and other centrally active agents, with predictable implications for exposure-dependent response and adverse effects. Individuals with higher-activity metabolizer genotypes may clear certain drugs rapidly, reducing the likelihood of achieving therapeutic concentrations, whereas reduced-function genotypes can slow elimination and increase the probability of supratherapeutic exposure. These relationships demonstrate how CYP450-driven variability can materially alter pharmacokinetics within defined metabolizer categories, and—depending on drug, dose, and clinical context—contribute to exposure-mediated adverse neuropsychiatric effects.

CYP450 metabolizer phenotypes describe functional metabolic capacity—often categorized as poor, intermediate, normal (extensive), and, where applicable, rapid/ultrarapid—based on measured or inferred enzyme activity rather than genotype alone. Genotype provides a baseline determinant of expression and function, but observed capacity reflects the integrated output of genetic variation, developmental stage, organ maturation, and dynamic modifiers such as inflammation and drug-drug interactions. In early infancy, ontogeny can dominate absolute clearance and may reduce the practical separation between genotype-predicted phenotype categories for many CYPs; accordingly, genotype is treated as one contributor to CYP capacity and interpreted against age-binned developmental expectations and state-dependent modifiers (including cytokine-mediated phenoconversion).

**Figure 1:**
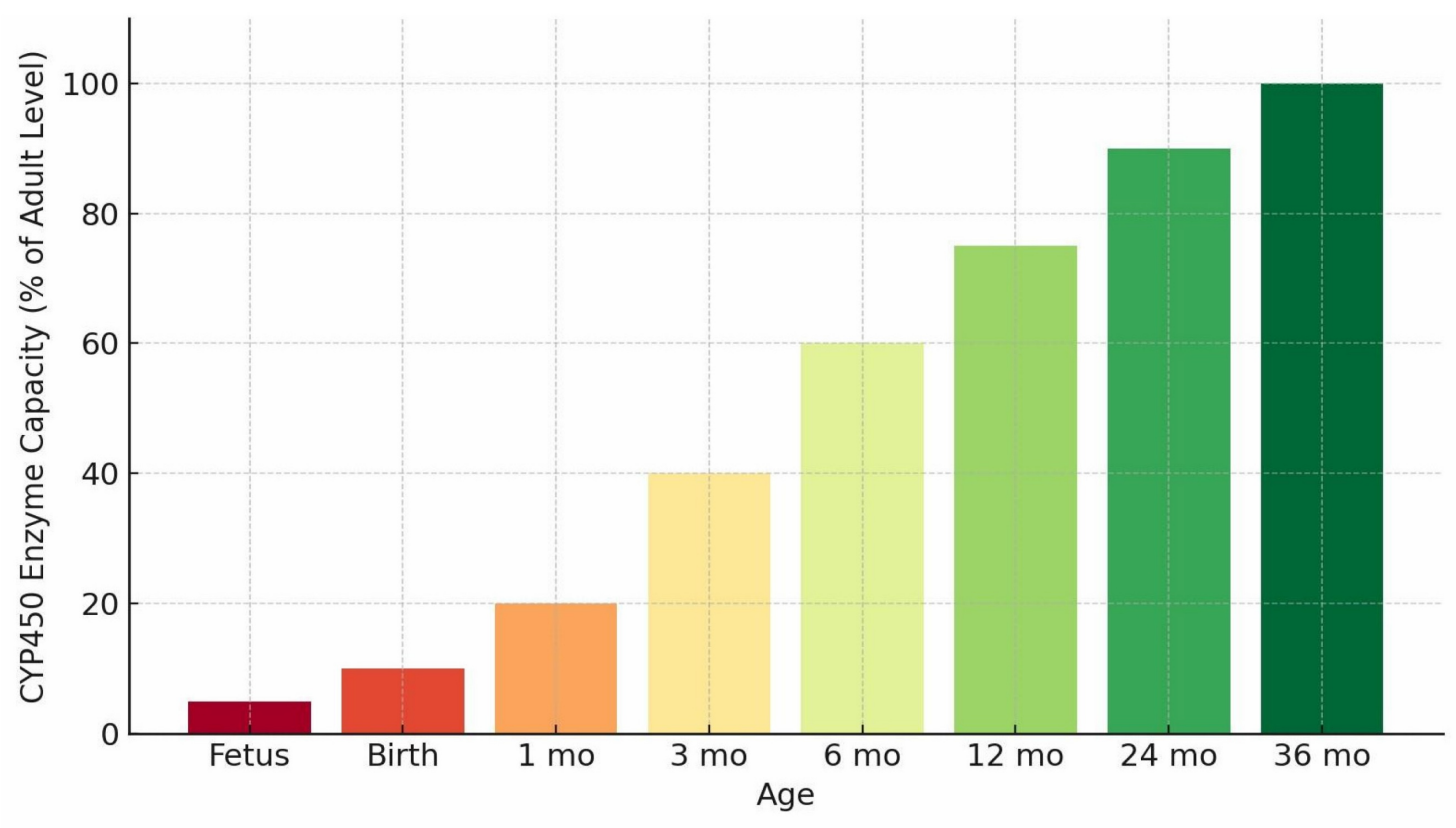
** Developmental Trajectory of CYP450 Enzyme Capacity.** Caption: Conceptual bar chart illustrating age-related increases in hepatic CYP450 metabolic capacity from the fetal period through early childhood. The trajectory reflects synthesized, aggregate ontogenic expression and activity patterns across major CYP450 isoforms and depicts relative functional capacity rather than direct enzymatic measurements or pharmacokinetic parameters.

**Figure 2:**
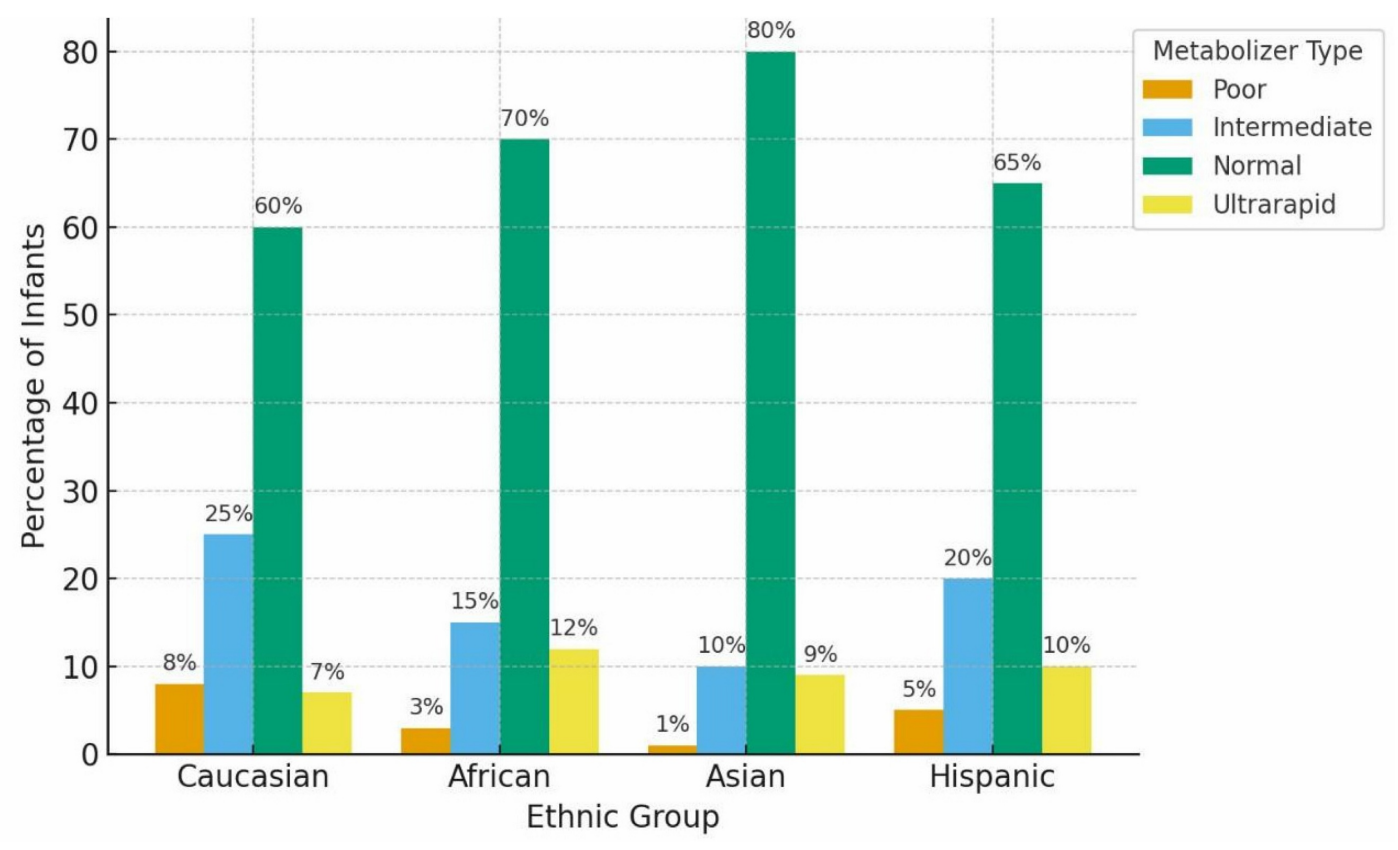
**Distribution of Functional CYP450 Metabolizer Phenotypes Across Representative Populations.** Caption: Conceptual population-level distributions of CYP2D6 and CYP2C19 metabolizer phenotypes (poor, intermediate, normal, and ultrarapid) across major ancestral groups. Values summarize well-established frequency patterns reported in pharmacogenomic reference cohorts and represent expected phenotype distributions prior to modification by developmental immaturity, inflammation-mediated phenoconversion, or environmental exposures.

Metabolic capacity is markedly reduced early in life because CYP ontogeny is isoform-specific and developmentally regulated, with maturation timelines that extend from the neonatal period through early childhood. Preterm infants exhibit especially low expression of several clinically relevant CYP isoforms and may also demonstrate delayed renal elimination, compounding constraints on clearance 4,5. The developmental trajectory of oxidative metabolic capacity is summarized in Figure 1. During periods of reduced baseline capacity, inherited variation in genes such as CYP2D6 and CYP2C19 can contribute to interindividual differences in drug clearance and exposure; however, in early infancy the practical separation between genotype-predicted metabolizer categories is often attenuated and depends on isoform, substrate, and developmental stage. Population-level distributions of CYP2D6 and CYP2C19 metabolizer categories across major ancestral groups are shown in Figure 2, illustrating how inherited variability may shape clearance potential across populations and may account for a larger fraction of interindividual variability when overall capacity is developmentally constrained.

Additional developmental variability arises from the neonatal transition within the CYP3A family, including the postnatal decline of fetal CYP3A7 and increasing expression of CYP3A4 and CYP3A5, which together contribute to changing CYP3A-mediated metabolic capacity over the first months of life 2-8. Although overall CYP3A activity is relatively low in early infancy, interindividual variability reflects time-varying developmental regulation superimposed on inherited genetic differences that persist throughout life. For example, CYP3A5 expression is more common among individuals with African ancestry, whereas CYP3A4 predominates in many individuals with European or Asian ancestry; these ancestry-linked patterns are present at all ages, but their practical impact may be accentuated during infancy when baseline capacity is developmentally constrained.

Early-life CYP450-mediated metabolism therefore operates at a fraction of adult capacity due to delayed isoform expression, limited hepatic metabolic throughput, evolving renal elimination, and context-dependent suppression of enzyme activity during systemic inflammation 2-8. These constraints intersect with inherited polymorphisms and exposure context—particularly medication exposures and other exogenous substrates that share metabolic pathways. Observational studies comparing outcomes across gestational age groups and exposure contexts 9 illustrate the broader principle that developmental immaturity and interindividual variability can contribute to heterogeneous vulnerability under certain conditions and in susceptible subgroups, even when population-averaged effects appear modest.

While CYP450-related susceptibility to adverse reactions from poorly metabolized psychotropic medications is well documented in later developmental periods 10,11, the extent to which early-life metabolic immaturity and context-dependent suppression intersect with exposure patterns to shape downstream outcomes remains incompletely characterized in infancy despite clearer evidence for exposure variability later in life. Accordingly, this review treats early-life metabolic vulnerability as hypothesis-generating, emphasizing mechanistic constraints and plausible interaction pathways rather than asserting causal links to specific infant clinical syndromes.

### The Three-Axis Convergence Framework (TCF)

**Figure 3:**
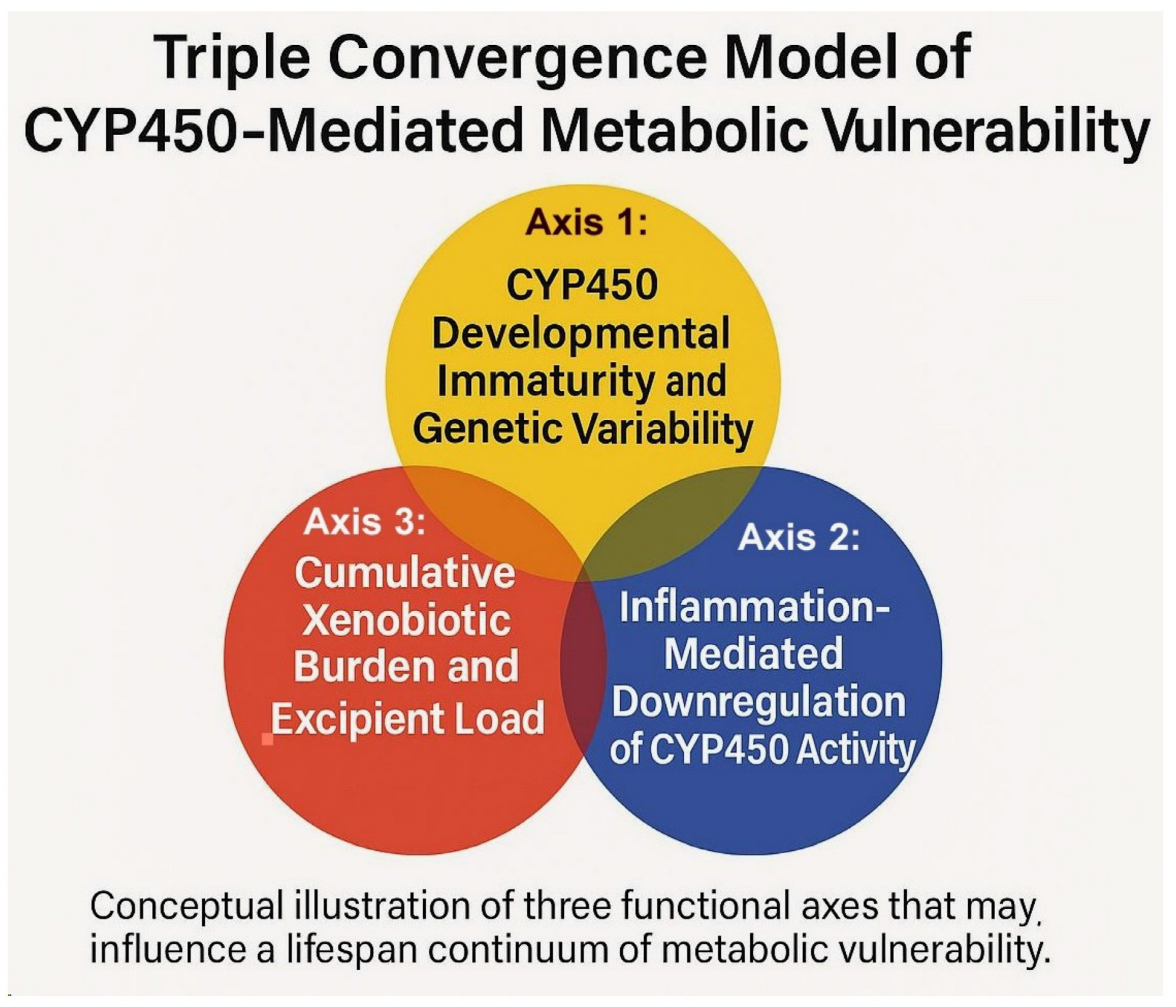
Three-Axis Convergence Framework (TCF) of CYP450-Mediated Metabolic Vulnerability. Caption: Diagram of the Three-Axis Convergence Framework (TCF), showing interactions among Axis 1 (developmental and genetic CYP450 capacity), Axis 2 (cytokine-mediated modulation of metabolism), and Axis 3 (cumulative xenobiotic and excipient load) as interacting determinants of metabolic vulnerability.

The Three-Axis Convergence Framework (TCF) conceptualizes metabolic vulnerability as the interaction of three analytically distinct but biologically coupled domains: Axis 1 (developmental and genetic reserve limits), reflecting isoform ontogeny and genotype-dependent variability; Axis 2 (immune-cytokine modulation and phenoconversion), reflecting inflammation-associated suppression of enzyme activity; and Axis 3 (exposure/disposition context), reflecting substrate inputs arising from medications and environmental xenobiotics that may compete for, induce, or inhibit metabolic pathways (Figure 3). Although depicted separately, these axes can converge within an individual and, under certain conditions, jointly narrow functional clearance capacity while increasing substrate pressure. Axis 1's developmental component diminishes with maturation, whereas genotype-dependent variability and state/exposure modulation (Axes 2-3) can remain relevant throughout life. Subsequent sections examine each axis and outline plausible convergence scenarios relevant to early-life physiology.

### Aggregation Artifacts from Unmodeled Heterogeneity

Population-level analyses may obscure clinically meaningful subgroup patterns when key modifiers of metabolic capacity—such as developmental stage, inflammatory status, and metabolizer category—are not measured or modeled directly. Unmodeled heterogeneity can attenuate associations, mask effect modification, or yield mixture-driven estimates that differ from subgroup-specific relationships. This motivates using metabolic variables as prespecified stratifiers and effect modifiers in epidemiologic study designs (i.e., measuring and modeling developmental stage, inflammatory markers, and metabolizer status), particularly in early infancy where baseline capacity is reduced and context-dependent phenoconversion may vary between individuals. This issue is revisited in the Discussion.

### Multidisciplinary Synthesis and Scope of This Review

This narrative review synthesizes evidence from pharmacogenetics, developmental pharmacology, toxicology, and developmental immunology to clarify how early-life CYP450 immaturity and ongoing modifiers—including inherited metabolic variability, immune-cytokine modulation, and exposure/disposition context—may shape metabolic vulnerability across the lifespan. Epidemiologic studies are included to contextualize observational findings and illustrate how metabolic constraints may manifest across populations. Emphasis is placed on developmental mechanisms and enzyme-environment interactions rather than on quantitative effect estimation, consistent with the hypothesis-generating intent of the early-life components of the TCF and the established evidence base in later-life psychotropic pharmacogenetics.

Building on the conceptual structure of the TCF, this review organizes mechanistic findings into recurring analytic domains (CYP450 capacity; immune/cytokine activity; redox balance/energetics; xenobiotic and metal exposure markers; and downstream neurochemical measures where available) to support structured interpretation and to highlight priorities for validation. Any multidomain interpretive schema is presented as preliminary and research-oriented, intended to inform future studies assessing feasibility, measurement stability, and reproducibility—particularly in early-life contexts where developmental physiology and timing strongly influence biomarker interpretability.

## 2. Methods

This narrative review synthesizes evidence from pharmacogenetics, developmental pharmacology, toxicology, immunology, and epidemiology to examine how CYP450 ontogeny, inherited variability, inflammatory phenoconversion, and exposure/disposition context may influence metabolic vulnerability. The review distinguishes two evidence tiers: (i) well-characterized clinical pharmacogenetic relationships in later life—particularly involving CYP2D6/CYP2C19—where genotype/phenotype differences predict measurable variation in drug exposure with documented links to clinical response and tolerability; and (ii) hypothesis-generating early-life mechanistic extensions, where clearance constraints and exposure/disposition context represent biologically plausible interaction pathways but direct empirical testing in infancy remains limited.

A comprehensive literature search was conducted using PubMed, Scopus, and Google Scholar for studies published between January 1990 and October 2025, with particular emphasis on literature from the past 10-15 years. Search terms included: CYP450, pharmacogenetics, pharmacokinetics, infant metabolism, cytokines, interleukin-6 (IL-6), CYP3A4/5, CYP2D6, CYP2C19, neurodevelopment, SIDS, phenoconversion, and vaccine adjuvants or excipients. Publications were included if they met at least one of the following criteria: (1) provided mechanistic, ontogenic, or quantitative data on CYP450 development, activity, or variability; (2) described genetic polymorphisms influencing CYP450 function or metabolic phenotype; (3) reported pharmacokinetic, toxicologic, or redox-related data relevant to xenobiotic, adjuvant, or excipient disposition; (4) examined cytokine-mediated modulation of drug-metabolizing enzymes; or (5) linked metabolic, inflammatory, or oxidative-stress pathways to neurodevelopmental or neurophysiologic outcomes.

Eligible sources included peer-reviewed original research, systematic reviews, meta-analyses, authoritative pharmacogenomic guidelines, and major regulatory or governmental reports published in English. References were cross-checked to identify additional studies meeting inclusion criteria. Where human infant data were unavailable—particularly regarding CYP-excipient interactions—validated modeling studies and toxicologic assessments from agencies such as the Food and Drug Administration (FDA), the Agency for Toxic Substances and Disease Registry (ATSDR), and the European Food Safety Authority (EFSA) were used to address targeted evidence gaps.

Extracted information was organized thematically across the three functional axes of the TCF. The narrative synthesis considered mechanistic plausibility, basic consistency across study types, and alignment with developmental physiology and available clinical or epidemiologic observations. Evidence was then mapped to five analytic domains—CYP450 capacity, immune/cytokine load, redox balance/energetics, neurochemical integrity, and xenobiotic/metal burden—to define the proposed Metabolic Vulnerability Index and integrated postmortem framework. Operational details of specimen validity, scoring, normalization procedures, and interpretive thresholds are provided in the appendices.

Use of AI Tools: ChatGPT (OpenAI) was used solely to assist with language editing and organization of manuscript text. No data generation, analysis, or interpretation was performed using AI tools.

## 3. Results / Evidence Synthesis

Evidence gathered through this narrative synthesis was organized across three functional axes describing CYP450-mediated metabolic vulnerability in early life: (1) developmental/genetic reserve limits, (2) immune-cytokine modulation of metabolism (phenoconversion), and (3) exogenous burden (xenobiotics, excipients, and metals). These axes form the mechanistic foundation of the TCF, illustrating how constrained enzymatic reserve, inflammatory signaling, and substrate burden can intersect during sensitive developmental windows. All primary citations supporting Tables 1-5 are provided with the tables.

### 3.1 Functional Axis 1—Developmental/Genetic Reserve Limits

Across developmental pharmacology, pharmacogenetics, and clinical cohort studies, the evidence summarized in Table 1 consistently supports Functional Axis 1 as a major source of constrained and heterogeneous metabolic reserve in early life. Human pharmacokinetic studies demonstrate markedly reduced oxidative clearance in neonates and young infants across multiple CYP isoforms, with particularly pronounced limitations in preterm populations 2-5,14. Within this developmentally constrained baseline, inherited variability in enzymes such as CYP2D6, CYP2C19, and CYP3A family members contributes to measurable interindividual differences in exposure for select substrates, even when absolute clearance remains low 7,12,13.

Importantly, multiple clinical and pharmacogenomic studies link reduced CYP-mediated clearance to higher drug concentrations and increased susceptibility to dose-related intolerance or adverse effects in later developmental periods, establishing a validated exposure-response framework for inherited metabolic variability 15-22. While phenotype separation may be attenuated in early infancy due to uniformly low expression, the same genetic determinants persist across development and become increasingly expressed as clearance capacity matures. Collectively, these findings support Axis 1 as a biologically grounded source of reserve limitation whose developmental and genetic components are well-characterized, reproducible across populations, and directly relevant to exposure sensitivity under constrained conditions.

### 3.2 Functional Axis 2—Immune-Cytokine Modulation of Metabolism (Phenoconversion)

Evidence across experimental, clinical, and translational studies demonstrates that systemic inflammation can reduce CYP450-mediated clearance through cytokine-dependent mechanisms that are isoform- and context-specific. Elevated inflammatory markers—most consistently IL-6 and CRP—have been associated with reduced activity or clearance of CYP3A4/5-, CYP2C19-, and CYP2D6-metabolized substrates in pediatric and adult populations experiencing clinically meaningful inflammatory states. A well-characterized clinical example is the IL-6-associated reduction in simvastatin clearance (a commonly used cholesterol-lowering statin), which is reversible following IL-6 receptor blockade with tocilizumab (an anti-IL-6 monoclonal antibody), supporting a direct cytokine-mediated effect on CYP3A activity 24.

Across studies, individuals with genotypes predicting normal metabolic capacity may transiently exhibit reduced functional clearance during inflammatory states, a phenomenon widely described as phenoconversion. Experimental hepatocyte models and human pharmacokinetic studies consistently demonstrate downregulation of CYP transcription and activity in response to pro-inflammatory cytokines, with variability by isoform, illness severity, and clinical setting 25-33. These findings establish immune-mediated modulation of CYP450 activity as a reproducible, state-dependent process rather than a fixed trait.

In the context of the TCF, Axis 2 functions as a dynamic modifier of effective metabolic capacity that can compound baseline reserve limits (Axis 1) and interact with exposure/disposition context (Axis 3). In early infancy—where baseline CYP450 capacity is developmentally constrained—systemic inflammation represents a plausible mechanism for further narrowing clearance margins when competing substrates are present, although direct infant-specific interaction studies remain limited.

Representative clinical, translational, and mechanistic evidence supporting Axis 2 is summarized in Table 2, which documents cytokine-associated modulation of CYP activity across multiple isoforms and developmental stages, while delineating the contexts in which such effects have been observed.

### 3.3 Functional Axis 3—Exogenous Exposure Context (Medications, Environmental Xenobiotics, Formulation Constituents, Metals)

Infants encounter a diverse range of exogenous inputs early in life, including nutrients, medications, and environmental chemicals. The systemic handling of these exposures depends on compound-specific factors such as dose, route, formulation, and timing, and may involve hepatic biotransformation, conjugation and transport processes, and/or renal elimination. Because these clearance pathways are developmentally regulated in early infancy, exposure context can influence disposition particularly when baseline CYP450 capacity is reduced (Axis 1) or transiently modulated by systemic inflammation (Axis 2).

Formulation constituents represent one component of this exposure context, but their relevance varies substantially by chemical class, concentration, route of administration, and disposition pathway. Accordingly, Axis 3 is framed to distinguish constituents with established systemic exposure and involvement of defined clearance pathways from those that are inert, minimally absorbed, or eliminated largely unchanged. Table 3 provides a descriptive inventory of selected formulation constituents present in routinely used infant immunizations in the 2025 U.S. schedule, summarizing disposition context (e.g., CYP-mediated, primarily conjugative, or largely non-CYP/renal) and explicitly noting where evidence is indirect, context-dependent, or does not demonstrate systemic relevance under typical exposure conditions. Notably, most listed constituents have no established direct CYP450 metabolism; where relevance is discussed, it is typically through non-CYP pathways (e.g., conjugation, hydrolysis, renal elimination) or indirect mechanisms rather than demonstrated CYP substrate competition at routine doses.

Within the Three-Axis Convergence Framework, Axis 3 is therefore treated as an exposure-context axis rather than a presumption of toxicity or metabolic overload. Its purpose is to characterize how the presence and timing of exogenous substrates—particularly those sharing disposition pathways—may contribute to variability in elimination under developmentally constrained or inflammation-modulated conditions, and how such substrates may interact with Axes 1 and 2 to shape effective metabolic reserve. This framing emphasizes dose-, route-, and kinetics-dependent relevance and avoids treating “excipients” or “metals” as a uniform or inherently cumulative class.

Evidence from neonatal and pediatric pharmacology demonstrates that certain exogenous constituents can become clinically relevant in specific contexts, particularly during sustained or high-dose exposure (e.g., continuous infusions or repeated therapeutic dosing), where clearance pathways may be rate-limiting and interindividual variability is pronounced. Table 4 summarizes representative experimental and clinical findings describing how selected exogenous constituents and metals intersect with disposition pathways (e.g., oxidation-conjugation balance, transporter dependence) and, in some settings, redox-related biology in early life. These findings are presented to delineate pathway intersections and measurement considerations rather than to assert established clinical harm.

Collectively, Axis 3 provides a structured approach for describing exogenous exposure context that may compound developmental reserve limits (Axis 1) and inflammation-associated modulation (Axis 2). The following section examines how these three axes can co-occur and interact during sensitive developmental windows.

### 3.4 Functional Interactions Within the TCF

The three axes of the TCF are analytically distinct but biologically coupled. In early life, developmental and genetic reserve limits (Axis 1) define baseline clearance capacity, while immune-cytokine signaling (Axis 2) can dynamically modulate drug-metabolizing enzymes and transport processes. Exogenous exposure context (Axis 3) can further influence observed disposition by introducing substrates that require shared clearance pathways (e.g., oxidation-conjugation balance, transporter dependence, renal elimination), particularly during periods when baseline capacity is developmentally constrained or transiently reduced by illness.

Within this synthesis, the TCF is used to describe how the co-occurrence of developmental immaturity, inflammation-associated modulation, and exposure context may jointly narrow effective metabolic reserve in a given physiologic setting. The framework is explicitly intended to support mechanistically stratified interpretation of heterogeneous exposures and states, rather than to imply causal attribution for any specific clinical outcome.

Table 5 integrates evidence across Axes 1-3 and highlights points of convergence on shared biologic processes relevant to systemic handling and physiologic tolerance, including redox balance, disposition pathway overlap, and cytokine-associated suppression of clearance. Additional illustrative examples of Axis interactions across infancy, adolescence, and adulthood are provided in Appendix B to contextualize these mechanisms across developmental stages; these examples are conceptual and are intended to complement—rather than extend—the evidence summarized in Table 5.

### 3.5 Synthesis and Framework Implications

The synthesized evidence indicates that early-life metabolic instability may arise when developmental/genetic reserve limits (Axis 1), immune-cytokine modulation (Axis 2), and exogenous exposure context (Axis 3) overlap during sensitive windows. For example, an infant with developmentally and/or genetically reduced CYP capacity (Axis 1) who experiences illness-related cytokine elevation (Axis 2) and concurrent exposure to compounds requiring hepatic or redox handling (Axis 3) may transiently exceed available clearance capacity. Under such conditions, constrained reserve may influence downstream systems—including neurochemical balance, autonomic regulation, or arousal pathways—particularly when multiple factors converge.

Depth of analytic evaluation within the TCF is explicitly conditional, with enhanced measures applied only when specimen validity, analytic feasibility, and domain-level findings warrant further resolution; this structure is intended to preserve rigor while avoiding indiscriminate testing.

Across the literature, vulnerability appears to exist along a continuum shaped by developmental stage, genotype, inflammatory state, and exposure timing. While each axis can contribute independently, their overlap may narrow physiologic reserve more than any single axis alone. Importantly, convergence does not imply predictable outcomes or deterministic effects; rather, it highlights conditions under which metabolic stress may be amplified, especially in infants with prematurity, inherited variability, or heightened inflammatory responses.

Consistent with this interpretation, the synthesis across Axes 1-3 is explicitly hypothesis-generating rather than conclusive. Several mechanistic intersections—particularly those involving early-life cytokine-mediated suppression and exposure-dependent clearance constraints—remain underexplored in empirical studies. The current findings delineate biologically plausible pathways and evidence convergence, while underscoring the need for targeted investigation to clarify prevalence, interaction strength, and subgroup specificity. Within this context, the TCF serves as a conceptual structure for identifying where key evidence gaps persist and where focused measurement and study design may be most informative.

Appendices C-E operationalize the TCF by defining the enhanced postmortem workflow, including specimen requirements and validity rules, analytic methods and QC conventions, and MVI/CMSP scoring and reporting logic (with a worked example). Appendices F-J provide supporting implementation materials, including age-matched CYP protein-abundance reference tables (Appendix F), operational roles (Appendix G), archetype lookup tables and referral rows (Appendix H), design rationale and limitations (Appendix I), and standardized terminology (Appendix J).

Together, these materials translate the TCF from a conceptual synthesis into a reproducible investigative framework suitable for systematic evaluation of Axis 1-3 interactions in unexplained infant deaths—without asserting causation or redefining certification criteria.

### 3.6 Consolidated Results of the Evidence Synthesis

The integrated synthesis of developmental pharmacology, pharmacogenetics, toxicology, immunology, and neuropathology supports organizing early-life metabolic vulnerability into three interacting axes: (1) developmental/genetic reserve limits, (2) immune-cytokine modulation of metabolism (phenoconversion), and (3) exposure/disposition context (xenobiotics, formulation constituents, and metals). Tables 1 and 2 summarize evidence supporting Axes 1 and 2, Table 4 summarizes evidence supporting Axis 3 in dose- and context-dependent settings, and Table 5 integrates cross-axis interactions and inference limits. Table 3 provides a descriptive disposition-context inventory for selected formulation constituents in the 2025 U.S. infant immunization schedule.

**Figure 4:**
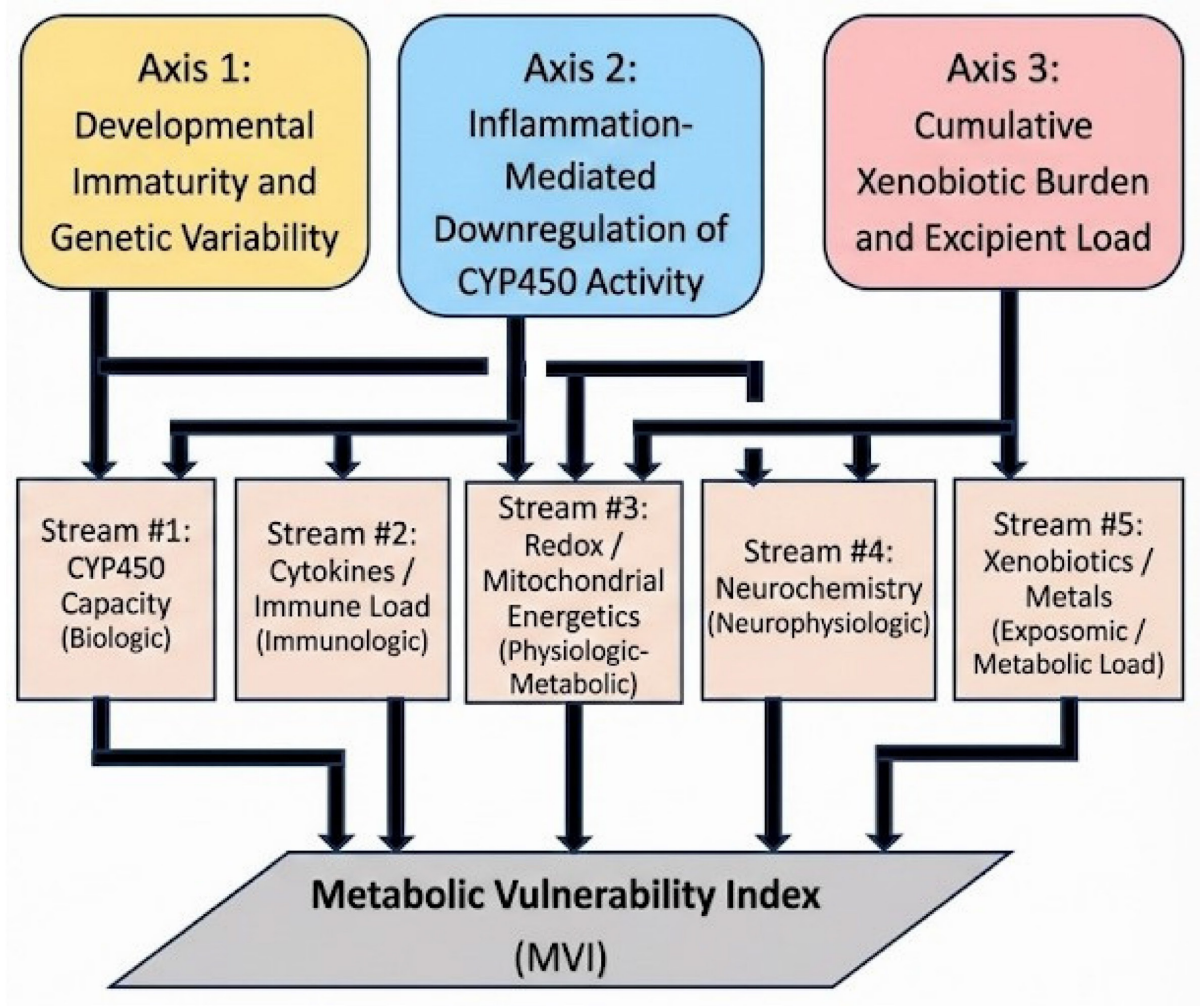
** The Mechanistic Axes of the TCF and Their Convergence upon Five Analytic Streams.** This figure depicts the three mechanistic axes of the TCF—developmental immaturity and genetic variation (Axis 1), inflammation-mediated suppression of metabolism (Axis 2), and cumulative xenobiotic or excipient load (Axis 3)—and their convergence upon the five analytic streams comprising the Metabolic Vulnerability Index (MVI). The diagram provides a conceptual synthesis of how upstream mechanistic pressures map onto distinct operational domains of metabolic vulnerability in early infancy.

For operational translation, this synthesis is mapped onto five measurable analytic domains that align with these axes and are intended to support mechanistic characterization without implying causation for any specific clinical outcome: CYP450 capacity (Domain 1), immune/cytokine load (Domain 2), redox balance/energetics (Domain 3), neurochemical integrity (Domain 4; modifier), and xenobiotic/metal burden (Domain 5; modifier/exposure context). This domain mapping and its relationship to the Three-Axis Convergence Framework (TCF) are summarized in Figure 4 and operationalized in Appendices C-E.

Consistent with this structure, Domains 1-3 are treated as the primary core domains for describing constrained reserve and state-dependent modulation, whereas Domains 4-5 provide downstream and exposure-context modifiers that support descriptive stratification when interpreted within standard forensic criteria. The corresponding domain scoring logic, safeguards, and reporting conventions are defined in the appendices. All primary citations are provided with Tables 1-5.

## 4. Discussion

This review does not propose new enzymatic mechanisms, nor does it assert causal explanations for unexplained infant deaths. Instead, its central contribution is the integration of well-established metabolic, immunologic, and toxicologic evidence into a structured interpretive framework that addresses persistent gaps in early-life mechanistic evaluation—particularly in postmortem contexts where standard investigations often lack physiologic resolution.

Across developmental pharmacology, pharmacogenetics, immunology, and toxicology, the literature consistently demonstrates that (i) metabolic capacity in early infancy is constrained and heterogeneous, (ii) immune activation can dynamically suppress clearance pathways, and (iii) exposure and disposition context determines whether such constraints are physiologically relevant. What remains poorly characterized is how these factors co-occur within individuals, how frequently they converge, and whether reproducible multidomain patterns consistent with constrained reserve can be identified using available postmortem measurements. These gaps are not primarily conceptual; they are methodological.

The Three-Axis Convergence Framework (TCF) addresses this limitation by organizing heterogeneous evidence into a minimal set of interacting mechanisms that can be evaluated empirically without presuming causation. The five analytic domains derived from this synthesis—CYP450 capacity, immune/cytokine load, redox balance/energetics, neurochemical integrity, and xenobiotic/metal burden—represent non-overlapping streams through which metabolic stress may be expressed biologically. Domains 1-3 capture core reserve limits and state-dependent modulation, while Domains 4-5 provide downstream and exposure-context modifiers that support interpretive coherence rather than driving classification.

Within this structure, the Metabolic Vulnerability Index (MVI) and the Cytokine-Metabolic Suppression Profile (CMSP) serve distinct but complementary roles. The MVI provides an ordinal, domain-based summary of multidomain constraint without weighting or causal inference, while the CMSP functions as an internal coherence check for immune-metabolic interaction patterns. Archetype classification further translates domain convergence into mechanistically interpretable profiles, enabling structured comparison across cases while preserving uncertainty and avoiding etiologic claims.

A key advance of this work is the translation of narrative synthesis into a practical postmortem workflow. By specifying specimen validity rules, analytic QC constraints, normalization strategies, and reporting logic, the framework enables selective, context-dependent depth of investigation within the primary medicolegal autopsy—rather than as a secondary or exploratory exercise. This approach acknowledges real-world constraints while creating a standardized pathway for evaluating whether constrained metabolic reserve is plausibly present in cases that remain unexplained after routine investigation.

Importantly, the framework is explicitly hypothesis-generating. Its purpose is not to redefine cause of death, but to determine whether reproducible, biologically coherent multidomain patterns can be identified across cases—and whether such patterns warrant further study. Priority next steps include independent validation of scoring reliability, cross-laboratory reproducibility, and assessment of archetype stability across populations and investigative settings.

**Practical feasibility and implementation considerations.** The TCF/MVI framework is intentionally designed to be modular and staged rather than prescriptive. While several components (e.g., comprehensive toxicology, IL-6/CRP measurement, and selected genetic testing) are technically feasible in many forensic or clinical laboratories, others—particularly redox/energetics anchors and optional neurochemical measures—are more sensitive to pre-analytic variability, postmortem interval, and assay standardization. Accordingly, implementation is best approached as a phased, consortium-oriented research-to-implementation pathway, prioritizing domains with higher analytic robustness and clearer interpretive value and expanding only after scoring reliability, cross-laboratory reproducibility, and specimen-handling consistency are demonstrated. This framing acknowledges real-world resource constraints while providing a standardized roadmap for systematic evaluation of metabolic vulnerability in cases that remain unexplained after routine investigation.

In this way, the TCF, MVI, and CMSP together provide a structured response to long-standing mechanistic uncertainty in early-life investigation: not by asserting new causes, but by enabling systematic measurement, disciplined interpretation, and transparent identification of evidence gaps where targeted research may be most informative.

### 4.1 Example Convergence Scenario (Illustrative, Non-Etiologic)

One empirically grounded convergence scenario is early infancy, when CYP3A-family capacity is developmentally low and variable, and clinically meaningful inflammation can further suppress CYP activity (phenoconversion). Under these conditions, exposure/disposition context becomes more relevant because the same dose/route/timing can yield different effective clearance margins across infants. This example is presented as a mechanistic illustration of Axis interaction, not as a claim about any specific outcome.

### 4.2 Exposure Context, Aluminum, and the Role of Uncertainty

The inclusion of aluminum-containing formulations and related epidemiologic literature in this framework is not intended to challenge the established public-health consensus regarding vaccine safety or effectiveness. For descriptive exposure context, aluminum content and timing across the U.S. childhood immunization schedule are summarized in Table 6, without implying toxicity, cumulative burden, or causation. Rather, it reflects a methodological decision to treat *all* recurrent early-life exposures—therapeutic, environmental, or preventive—as part of a unified exposure-disposition context when evaluating metabolic reserve under constrained developmental conditions. Aluminum-based adjuvants are included because they are quantitatively defined, temporally anchored exposures with published pharmacokinetic modeling, making them suitable for structured disposition-context description and sensitivity-to-assumption analysis—rather than for causal inference 34-35.

Similarly, epidemiologic studies reporting subgroup-level associations or temporal patterns (e.g., Mawson et al. 9) are cited to illustrate heterogeneity in observational findings and the limits of population-averaged inference when key biologic modifiers are not measured, not as evidence of harm. Such studies underscore the limitations of population-averaged analyses when metabolic capacity, immune state, and exposure timing are not directly measured or stratified. Within the Three-Axis Convergence Framework, these uncertainties motivate *measurement and stratification*, not presumption of toxicity or etiologic conclusions. Accordingly, aluminum and other formulation constituents are treated strictly as exposure-context variables whose relevance depends on dose, route, timing, and individual physiologic state, and whose inclusion supports disciplined evaluation of evidence gaps rather than causal attribution.

## 5. Limitations

This narrative review integrates evidence from developmental pharmacology, pharmacogenetics, toxicology, immunology, and observational research to propose a hypothesis-generating organizational framework rather than to establish causal relationships. Several limitations warrant emphasis.

First, while developmental CYP450 ontogeny and inherited pharmacogenetic variability (Axis 1) are supported by extensive human data, evidence directly linking immune-mediated metabolic suppression (Axis 2) and exposure/disposition context (Axis 3) to defined clinical outcomes in early infancy remains limited and context-dependent. Many findings are substrate-, illness-, and timing-specific, and in early life it is often difficult to disentangle developmental regulation from state-dependent suppression in the absence of integrated biomarker data.

Second, the outcome categories discussed—including unexplained infant deaths and heterogeneous neurodevelopmental outcomes—are multifactorial and shaped by diverse genetic, developmental, clinical, and social determinants. Metabolic and inflammatory pathways therefore represent only one potential contributor within a complex etiologic landscape, and observational associations should not be interpreted as causal without prespecified study designs and mechanistic adjudication.

Third, several evidence streams informing Axes 2-3 and Domains 3-4 derive from heterogeneous sources, including in vitro studies, animal models, pharmacokinetic investigations, and limited clinical or postmortem observations. These measures vary in generalizability and are sensitive to timing, illness severity, specimen handling, and postmortem interval. Accordingly, the framework is not intended to estimate population-level risk, define thresholds, or assign individual-level causation, but to organize evidence, clarify measurement priorities, and identify areas requiring prospective validation and reproducibility testing.

Collectively, these limitations underscore the need for cautious inference and motivate future work emphasizing standardized measurement protocols, integrated datasets, and subgroup-aware study designs capable of evaluating biologic heterogeneity without overstating clinical or forensic interpretability.

## 6. Conclusions

This work introduces the Three-Axis Convergence Framework (TCF) and its operational translation, the Metabolic Vulnerability Index (MVI), as a structured, hypothesis-generating approach for organizing multidomain postmortem data in unexplained infant deaths. By integrating developmental/genetic reserve limits, immune-cytokine modulation, and exposure/disposition context into a unified analytic structure, the framework enables systematic evaluation of whether reproducible patterns consistent with constrained metabolic reserve are present under standardized measurement conditions.

The MVI is intended for evaluative use alongside routine medicolegal investigation, without redefining cause of death or implying causation. Its principal contribution is not diagnostic attribution, but the ability to identify convergent physiologic patterns, delineate inference limits, and clarify where mechanistic evidence is strongest versus where targeted investigation is most needed. In this way, the TCF, MVI, and CMSP together provide a structured response to long-standing mechanistic uncertainty in early-life investigation—not by asserting new causes, but by enabling systematic measurement, disciplined interpretation, and transparent identification of evidence gaps where targeted research may be most informative. Priority next steps include independent validation of scoring reliability, cross-laboratory reproducibility, and prospective assessment of pattern coherence across infant subgroups.

## 7. Future Directions

Future research should evaluate how developmental enzyme ontogeny, inflammatory signaling, and exposure context interact in early life using longitudinal, mechanistically informed designs. Targeted cohorts—particularly preterm, growth-restricted, and medically fragile infants—are well suited to characterize how CYP450 genotype, age-dependent metabolic capacity, and cytokine-associated phenoconversion relate to time-varying pharmacokinetics and physiologic state (including redox/energetics measures where analytically robust).

Prospective epidemiologic studies would benefit from incorporating metabolic heterogeneity through prespecified subgroup analyses and, where feasible, integration of pharmacogenomic and inflammatory biomarker data. These approaches can improve modeling of effect modification and reduce aggregation artifacts that arise when biologically distinct strata are pooled, thereby strengthening inference about subgroup variation without overstating causality.

In parallel, multicenter postmortem and translational studies should prioritize analytic harmonization to enable valid cross-site comparison of multidomain findings. Key needs include standardized specimen collection and handling, assay calibration and quality-control thresholds, reporting conventions, and inter-laboratory proficiency testing. Preregistered analytic plans, rigorous confounder control, and replication across independent medicolegal jurisdictions will be essential to determine which multidomain patterns are reproducible, how frequently they occur, and how they relate to measurable physiologic contexts (e.g., prematurity, intercurrent illness, and timing relative to clinical events). Over time, such work can support clearer interpretation boundaries for developmental immaturity versus state-dependent modulation and can guide which measurements add incremental value in cases that remain unexplained after routine evaluation.

## Figures and Tables

**Table 1:** ** Axis 1: CYP450 Developmental Immaturity and Genetic Variability — Evidence-Based Studies and Key Findings.** Summary of evidence supporting Axis 1, including developmental CYP450 immaturity and inherited pharmacogenetic variability, with clinical implications for drug exposure and response.

Ref.	Key Findings / Evidence Summary	Mechanistic Relevance	Applicable Outcomes
2-7	Neonates and young infants have reduced CYP450 activity, with isoform-dependent maturation of hepatic metabolism and renal elimination over time. Pharmacokinetic studies demonstrate age-dependent reductions in clearance and prolonged half-lives for selected CYP substrates.	Characterizes early-life developmental constraint on oxidative metabolism and clearance.	Age-dependent drug exposure; dosing sensitivity in early infancy; exposure-related adverse effects for select CYP substrates.
12-15	Pharmacogenomic studies define CYP2D6/CYP2C19 genotype-based metabolizer categories and document population-level allele variation contributing to predictable interindividual differences in drug exposure.	Quantifies inherited metabolic variability across populations and across the lifespan.	Drug-gene interaction risk; exposure variability for CYP2D6/CYP2C19 substrates; substrate-specific differences in tolerability/response.
14	Preterm infants demonstrate greater physiologic immaturity than term infants, including reduced hepatic metabolic capacity and renal elimination during early life, which can affect drug handling during physiologic stress.^a^	Identifies a subgroup in which developmental constraints on clearance may be amplified by prematurity-related physiology.	Preterm dosing complexity; increased sensitivity to exposure-related adverse effects in select therapeutic contexts.
15-18	Reduced-function CYP2D6/CYP2C19 genotypes are associated with higher psychotropic drug exposure and increased dose-related intolerance, often mitigated by dose adjustment or medication change.	Demonstrates exposure-mediated adverse effects as a function of reduced metabolic clearance for specific substrates.	Adverse drug reactions; dose intolerance; exposure-mediated adverse effects (drug- and context-dependent).
18-19	Some cohorts report that higher-activity metabolizer genotypes can be associated with lower drug concentrations and reduced response for selected antidepressants, consistent with rapid clearance in substrate-specific contexts; associations with clinical endpoints may vary by study design and confounding.	Illustrates that increased metabolic activity can reduce exposure and compromise efficacy for certain drugs depending on pathway reliance and dosing.	Treatment nonresponse for selected substrates; need for dose optimization/alternate agents; heterogeneous clinical associations in observational cohorts.
20-22	Pharmacogenetic reviews and the PRIME Care RCT support the clinical utility of CYP2D6- and CYP2C19-informed prescribing for certain antidepressants by reducing actionable drug-gene interactions and informing medication selection/dosing.	Establishes clinical actionability of inherited CYP variability for select drug classes.	Treatment optimization; reduction of actionable drug-gene interactions; potential reduction in exposure-related adverse effects.
23	Population-level data show increased psychotropic drug use in youth following major stressors, expanding exposure in groups with heterogeneous metabolic capacity.	Highlights how changes in prescribing patterns can increase the population impact of interindividual pharmacokinetic variability.	Expanded exposure to CYP substrates in youth; greater importance of dose selection and monitoring in heterogeneous populations.

^a^ Indirect support: Preterm infants exhibit broader physiologic immaturity (hepatic, renal, and immune), which can amplify variability in drug handling and illness-related modifiers during early life.

**Table 2:** ** Axis 2: Inflammation-Mediated Modulation of CYP450 Activity—Evidence-Based Studies and Key Findings.** Summary of experimental, clinical, and translational evidence describing cytokine-associated modulation of CYP450 enzymes and inflammation-related reductions in metabolic clearance.

Ref.	Key Findings / Evidence Summary	Mechanistic Relevance	Applicable Outcomes
25	Pediatric studies report associations between inflammatory states (often indexed by IL-6/CRP) and reduced activity/clearance of selected CYP3A and CYP2C substrates during clinically significant inflammation; magnitude varies by setting, substrate, and illness severity.	Supports cytokine-associated modulation of major CYP pathways in pediatric inflammatory contexts.	Illness-associated exposure variability; functional phenoconversion; potential for increased exposure/ accumulation for select substrates during inflammation.
26	CYP3A and CYP2C isoforms show selective sensitivity to pro-inflammatory cytokine conditions in experimental systems.	Demonstrates isoform-specific susceptibility to cytokine modulation.	Selective metabolic suppression in inflammatory contexts; variable clearance across substrates.
27	IL-6 and IL-1β reduce CYP2C, CYP2B6, and CYP3A4 mRNA in hepatocyte models.	Defines transcriptional mechanisms contributing to reduced metabolic capacity during inflammation.	Reduced clearance potential in inflammatory states (mechanistic support).
28	Review literature links infection/inflammation to altered drug-metabolizing enzyme activity and clinically meaningful changes in pharmacokinetics for some drugs.	Integrates cytokine-associated CYP modulation into clinical pharmacokinetics.	Drug-disease interactions; illness-associated reductions in clearance for selected substrates.
29	Critically ill children show reduced clearance of midazolam (a CYP3A substrate) in association with elevated inflammatory markers/cytokines.	Provides clinical evidence consistent with inflammation-associated phenoconversion during severe illness.	Exposure variability in critical illness; potential for drug accumulation for CYP3A substrates during systemic inflammation.
30	IL-6-associated CYP3A4 suppression is reversible with IL-6 pathway blockade in vitro.	Supports reversibility and pathway specificity of cytokine-associated modulation.	Reversible phenoconversion; cytokine-linked drug-disease interaction plausibility.
31	Immune ontogeny literature describes age-specific immune responsivity in early life and cytokine output during common immune challenges.	Provides developmental immune context; not sufficient alone to establish CYP suppression.ᵃ	Developmental immunology context; relevance when systemic inflammation is present.
32	Clinical and translational literature describes reduced clearance of some CYP-metabolized substrates during infection/inflammation across patient populations, with variability by isoform and clinical setting.	Confirms translation from mechanistic models to human pharmacokinetics in inflammatory states.	Illness-associated reductions in clearance for selected substrates; exposure-related adverse effect risk (context-dependent).
33	Reviews describe broader regulatory links between cytokine cascades and drug metabolism/disposition across CYP isoforms.	Establishes systemic context for inflammatory modulation of metabolism.	Framework support for phenoconversion; mechanistic context for drug-disease interactions.

^a^ Indirect developmental context: early infancy features evolving CYP ontogeny and distinct immune signaling; co-occurrence does not imply exposure-specific CYP suppression.

**Table 3:** ** CYP450-Relevant Disposition Context for Selected Formulation Constituents in the 2025 U.S. Infant Immunization Schedule (Birth-24 Months).** Selected formulation constituents are summarized descriptively by primary disposition pathway and evidence context. Inclusion is informational and does not imply toxicity, cumulative burden, or clinically meaningful CYP450 modulation under routine exposure conditions. Global notes (apply to all rows): (i) Mechanistic evidence often derives from non-vaccine, high-dose, or non-infant contexts and does not establish clinically meaningful effects under routine immunization exposure. (ii) Several constituents (e.g., aluminum salts, polysorbates) are not CYP-metabolized; any indirect links are context-dependent. (iii) Trace constituents should be interpreted relative to endogenous background and dose/route.

Vaccine	Excipients^a^	CYP450 relevance (descriptive)	Evidence context (coded)	Ref.
Hepatitis B (Engerix-B)	Aluminum hydroxide	Not a CYP substrate; not CYP-metabolized	(ii)	7,26-31,34-35
	Formaldehyde (residual)	Not primarily CYP-metabolized	(iii)	26-38
DTaP (Infanrix)	Aluminum hydroxide/ phosphate	Not a CYP substrate; not CYP-metabolized	(ii)	7,26-31,34-35
	Polysorbate-80	No established direct CYP metabolism	(ii)	37
	Formaldehyde (trace)	Not primarily CYP-metabolized	(iii)	36,38-39
	2-Phenoxy-ethanol	Predominantly conjugation; limited oxidative metabolism reported	(i)	36
	Glutaraldehyde (trace)	Not a typical CYP substrate	(i)	38
Hib (PedvaxHib)	Aluminum hydroxide	Not a CYP substrate; not CYP-metabolized	(ii)	7,26-31,34
	Formaldehyde	Not primarily CYP-metabolized	(iii)	36,38-39
PCV13 (Prevnar-13)	Aluminum phosphate	Not a CYP substrate; not CYP-metabolized	(ii)	7,26-31,34
	Polysorbate-20	No established direct CYP metabolism	(ii)	37
IPV (Ipol)	2-Phenoxyethanol	Predominantly conjugation; limited oxidative metabolism reported	(i)	36
	Formaldehyde	Not primarily CYP-metabolized	(iii)	36,38-39
Rotavirus (RotaTeq)	Polysorbate-80	No established direct CYP metabolism	(ii)	37
Influenza (Fluzone Quadrivalent, pediatric)	Triton X-100	No established CYP-specific metabolism	(i)	37
	Formaldehyde	Not primarily CYP-metabolized	(iii)	36,38-39
	Antibiotics (trace)	Not a CYP category; class-dependent elimination	(i)	28
MMR (M-M-R II)	Neomycin (trace)	Not a CYP category; class-dependent elimination	(i)	28
Varicella (Varivax)	Neomycin (trace)	Not a CYP category; class-dependent elimination	(i)	28
Hepatitis A (Havrix)	Aluminum hydroxide	Not a CYP substrate; not CYP-metabolized	(ii)	7,26-31,34
	Polysorbate-20	No established direct CYP metabolism	(ii)	37

^a^ Excipients without established CYP450 relevance or systemic disposition under typical exposure may be omitted for clarity.

**Table 4:** ** Axis 3: Exposure Context and Disposition Pathways for Selected Exogenous Constituents in Early Life—Evidence-Based Studies and Key Findings.** Evidence summarizing disposition pathways and dose/context dependence of selected exogenous constituents, including examples where sustained or high-dose exposure (primarily in therapeutic settings) produces clinically meaningful toxicity in neonates. Findings are presented to clarify pathway intersections and measurement considerations, not to infer causality.

Ref.	Key Findingsᵃ / Evidence Summary	Mechanistic Relevance	Applicable Outcomes (scope-limited)
37	Polysorbate 20/80 exhibit hydrolysis/ester exchange in some formulation contexts; mechanistic reports describe oxidative instability under specific conditions.	Illustrates that excipient behavior can be formulation- and context-dependent; does not establish CYP-mediated metabolism.	Formulation science context; dose- and setting-dependent relevance.
38	Formaldehyde is a reactive aldehyde; toxicologic literature describes cellular injury mechanisms at sufficiently high exposures.	Provides general mechanism background for aldehyde reactivity; clinical relevance depends on dose and endogenous handling.	Toxicology mechanism reference; not outcome-specific.
34	Pharmacokinetic modeling and related literature describe various aluminum kinetics and elimination assumptions under scenarios.	Context for disposition modeling; emphasizes route- and assumption-dependence rather than CYP involvement.	PK modeling context; exposure characterization.
35	Tracer-based work describes absorption and distribution kinetics for aluminum from specific sources/routes.	Illustrates that systemic handling may be gradual and route-dependent; not CYP-mediated.	Kinetic characterization; exposure context.
40	Surveys of neonatal medicines document frequent inclusion of excipients in therapeutic formulations.	Establishes that excipient exposure is common in neonatal drug formulations (often repeated dosing).	Exposure prevalence in neonatal therapeutics.
41	Reviews identify excipients of potential concern in neonatal formulations and emphasize context- and dose-dependent safety.	Supports structured excipient evaluation in neonatal therapeutics where exposure can be sustained.	Formulation risk assessment context; monitoring/selection considerations.
42	Reviews of propylene glycol in neonates describe toxicity risk under high or prolonged dosing with immature clearance.	Demonstrates a clinically established example of excipient toxicity driven by dose, duration, and limited neonatal metabolism.	Neonatal therapeutic toxicity risk (high-dose/prolonged exposure contexts).
43	Pediatric oral formulation reviews discuss excipient safety profiles and the importance of age-appropriate dosing/exposure limits.	Supports dose-dependent interpretation and heterogeneity across excipient classes.	Pediatric formulation safety context; dose sensitivity.
44	Historical clinical reports link benzyl alcohol exposure in preterm neonates to severe toxicity under specific dosing circumstances (“gasping syndrome”).	Canonical example that sustained excipient exposure can be clinically hazardous in vulnerable neonates.	Clinically established neonatal excipient toxicity (specific exposure circumstances).
36	Reviews of alcohol metabolism describe enzymatic pathways for ethanol oxidation and acetaldehyde handling, via ADH/ALDH.	Clarifies that ethanol handling is primarily dehydrogenase-mediated rather than CYP-driven in typical contexts.	Metabolic pathway background; substrate-specific disposition.
45	Clinical observations in ill neonates describe altered ethanol disposition and slower clearance compared with older children/adults.	Illustrates illness- and developmental-stage effects on clearance for select substrates.	Developmental PK variability in illness contexts.
39	ADH5/ALDH pathways are implicated in formaldehyde detoxification biology; endogenous handling is substantial relative to many trace exposures.	Supports pathway specificity for aldehyde detoxification; reinforces dose-context dependence.	Metabolic pathway background; interpretation framework.
46	Experimental literature explores transport/distribution of particulate materials under specific conditions and models.	Contextual mechanistic literature; generalizability depends on model, material form, and exposure conditions.	Mechanistic model context; hypothesis framing only.
47	Exposome/biomonitoring work describes early-life exposure complexity across chemical classes.	Provides population-level context motivating exposure characterization rather than single-agent inference.	Exposure characterization context; hypothesis generation for measurement strategies.

^a^ Findings describe biologic mechanisms and exposure pathways only; exposure presence does not imply toxicity or causation and must be interpreted by dose, route, duration, developmental stage, and illness context.

**Table 5:** ** Functional Interactions and Inference Limits Across TCF Axes.** Integrated literature illustrating Axis 1-3 interactions, pathway convergence, and inference limits in early-life physiology; inclusion does not imply causation.

Ref.^a^	Key Findings / Evidence Summary	Mechanistic Relevance	Applicable Outcomes (scope-limited)
48	Comprehensive review of vitamin D metabolism, including CYP enzymes involved in activation/inactivation (e.g., CYP27B1, CYP24A1) and pleiotropic developmental effects.	Illustrates that CYP-family enzymes extend beyond hepatic drug-metabolizing CYPs and participate in developmental physiology; supports careful boundary definition between drug-metabolizing CYPs and other CYP systems when mapping “Axis 1” biology.	Developmental physiology context; endocrine-metabolic pathway interpretation.
49	Review of blood-retinal barrier behavior under hypoxic-ischemic conditions and oxidative stress, including mechanisms of permeability changes and microvascular injury.	Provides general biologic context for how hypoxia/oxidative stress can influence microvascular barrier integrity; relevant as a downstream process that can covary with systemic illness and physiologic stress.	Barrier biology context; hypoxia/oxidative stress mechanisms (non-diagnostic).
50	Clinical, pathologic, and biomechanical analysis examining infant shaking/impact scenarios and injury mechanisms within modeled and observed parameters.	Demonstrates the role and limitations of biomechanical modeling in estimating force ranges; underscores that simulations alone do not measure physiology or establish etiology without clinical/pathologic correlation and confounding control.	Biomechanics + clinicopathologic correlation context; limits of model-only inference.
51	Anthropomorphic simulations compare falls, shakes, and impacts in infants, generating modeled ranges of rotational acceleration under different scenarios.	Highlights parameter sensitivity in simulations and the need to interpret modeled forces as plausibility bounds rather than physiologic measurements or case-level causal inference.	Biomechanics modeling context; parameter sensitivity/uncertainty.
52	Biomechanics-focused analysis discussing candidate injury mechanisms and interpretation within SBS-related literature.	Included as a contested biomechanics source highlighting the vulnerability of retrospective causal inference to modeling assumptions and interpretive disagreement.	Forensic interpretation context; controversy management and inference limits.
53	Postmortem studies in SIDS report brainstem serotonergic abnormalities in subsets of cases (e.g., receptor-related findings in medullary regions implicated in arousal).	Establishes a replicated neurobiologic correlate in SIDS literature; included here as a parallel vulnerability domain that should be interpreted as a correlate (not a demonstrated consequence of CYP-mediated metabolism).	SIDS neuropathology correlate; arousal/autoresuscitation vulnerability context.
54	Additional SIDS studies report serotonergic transporter-related differences in brainstem regions implicated in autoresuscitation and arousal.	Reinforces that SIDS research includes measurable neurobiologic correlates and supports careful separation of correlates from mechanistic causal claims.	SIDS neuropathology correlate; interpretive context (non-etiologic).
55	Early Neurology report evaluated temporal proximity patterns between DTP immunization and SIDS reports in historical datasets and raised a hypothesis of association.	Included as historical context illustrating limits of temporal proximity designs, which are sensitive to age overlap, selection bias, and reporting artifacts.	Historical epidemiology context; temporal association limitations (non-causal).
56	Early Pediatrics report assessed temporal association between DTP immunization and SIDS using a defined post-immunization window; included as historical context highlighting limitations of temporal clustering designs.	Illustrates methodological limits of temporal clustering analyses and the need for modern causal designs with prespecified confounding control.	Historical epidemiology context; limits of post-immunization temporal window analyses (non-causal).

^a^ Global note: Sources are heterogeneous (mechanistic, modeling, neuropathology, historical epidemiology) and are included to define measurement domains and inference limits, not to support etiologic claims.

**Table 6:** ** Aluminum Content of Vaccines in the U.S. Childhood Immunization Schedule (2025).** This table summarizes vaccines in the U.S. childhood immunization schedule that contain aluminum-based adjuvants, including infant and later childhood vaccines, to contextualize cumulative and life-course exposure. Infant vaccines are the primary focus of the present framework; later vaccines are included for completeness and comparative reference.

Vaccine	Adjuvant Type	Aluminum/ Dose (µg)^a^	Age of Administration (months)	Notes
Hepatitis B (HepB)	Aluminum hydroxide or phosphate	~250	Birth, 1-2, 6-18	Initiates aluminum exposure at birth; included in Mitkus model.
DTaP	Aluminum phosphate and/or hydroxide	170-625	2, 4, 6, 15-18	Multiple formulations; cumulative early exposure.
Hib	Aluminum hydroxide (some formulations)	~225	2, 4, 6, 12-15	Conjugate vaccines vary; some brands non-aluminum.
PCV13	Aluminum phosphate	~125	2, 4, 6, 12-15	Widespread use; four aluminum exposures in first year.
Hepatitis A (HepA)	Aluminum hydroxide	~250	12-23 (2 doses, ≥6 months apart)	Adds to cumulative body burden before two years.
HPV	Aluminum hydroxyphosphate sulfate	~225	9-12 years (3-dose series)	Beyond infant window; relevant for cumulative lifetime exposure.
MenACWY	Aluminum phosphate	~250	11-12 years, booster at 16	Not infant exposure, included for total adjuvant profile.

^a^ Aluminum content values are approximate and may vary by manufacturer and lot. Data compiled from CDC vaccine excipient summaries (2024-2025) 57.

**Table C1:** ** Approximate Postmortem Stability Windows.** Indicative post-collection handling targets and practical PMI ranges for selected biomarker domains.

Domain: Description	Test	Preferred specimen / matrix	Best within (PMI)	Max PMI (sampling)^a^
1: CYP450 Capacity	Targeted LC-MS/MS proteomics (MRM/SRM) preferred (pmol/mg)	Liver (standardized lobe: e.g. left lobe)	<24 h	≤48 h
2: Cytokine load	IL-6 immunoassay (pg/mL)	Femoral venous blood (not cardiac) → serum (preferred) or EDTA plasma	<24 h	≤48 h
	CRP (mg/L; not hs-CRP)	Femoral venous blood (not cardiac) → serum (preferred) or plasma	<24 h	≤48 h
3: Redox status	F2-isoprostanes (8-iso-PGF2α tissue; ng/g tissue, wet weight) LC-MS/MS or GC-MS/MS preferred (ng/g)	Liver (default; standardized lobe: e.g., left lobe); optional corroboration: Kidney cortex (standardized region)	<24 h	≤48 h
Endocrine/ Physiologic amplifier (contextual)	Vitreous glucose (mmol/L) + β-hydroxybutyrate (BHB) (mmol/L)	Vitreous humor (both eyes per SOP)	<24 h	≤72 h

**Table E1:** ** Conceptual Integration of Domains for MVI Scoring (Non-Procedural).** Overview of the five conceptual domains integrated in the MVI framework, including primary scored anchor measures and their contribution to overall vulnerability characterization.

Domain (conceptual)	Primary data integrated (scored anchor measures)	Contribution to MVI
Specimen foundation	Liver, brainstem, femoral blood, vitreous; ancillary tissues as available	Ensures interpretability and domain eligibility (Appendix C)
Domain 1: CYP450 capacity	Hepatic CYP capacity (isoform protein abundance preferred; activity may be reported when QC-qualified)	Quantifies metabolic reserve and reserve limitation
Domain 2: Cytokine load	IL-6 and CRP severity (femoral serum/plasma)	Captures immune activation magnitude compatible with metabolic suppression
Domain 3: Redox balance	Tissue F2-isoprostanes (8-iso-PGF2α; liver default; kidney optional corroboration)	Captures oxidative stress burden (lipid peroxidation)
Domain 4: Neurochemical integrity	Brainstem neuropathology ± serotonergic IHC (SERT/TPH2) as operational anchors; frozen neurochemistry optional	Captures autonomic/arousal circuitry vulnerability signals (modifier domain)
Domain 5: Xenobiotic/metal burden	Comprehensive toxicology ± metals (ICP-MS; speciation/confirmation conditional)	Documents exogenous burden as exposure context/modifier; supports overload patterns when paired with Domains 1-3

**Table E2:** ** CMSP Scoring Table (Ordinal Bins 0-3).** Ordinal severity bins and calculation logic for CMSP Core and final CMSP scoring based on cytokine and redox anchor measures, with vitreous markers reported as contextual physiologic indicators.

Component: Measurement (units)	Normal (Score 0)	Mild (Score 1)	Moderate (Score 2)	Severe (Score 3)
Cytokine: IL-6 immunoassay (pg/mL)	≤30	30-80	80-300	>300
Cytokine: CRP (mg/L; not hs-CRP)	≤10	10-40	40-150	>150
Redox: F2-isoprostanes (8-iso-PGF2α; × Ref.)	≤1×	1-2×	2-4×	>4×
Physiologic: Vitreous β-hydroxybutyrate (mmol/L) *(contextual)*	≤2.5	2.5-5	5-10	>10
Physiologic: Vitreous glucose (mmol/L) *(contextual)*	≤10	10-15	15-25	>25

**Table E3:** ** Vitreous Glucose and β-Hydroxybutyrate Contextual Pattern Guide.** Interpretive pattern framework for vitreous glucose and BHB as contextual physiologic amplifiers; non-diagnostic and not incorporated into CMSP or MVI scoring.

Vitreous glucose^a^ (ordinal)	Vitreous BHB^a^ (ordinal)	Pattern label (contextual)	What it can add (context only; non-diagnostic)
0-1 (normal-mild)	0-1 (normal-mild)	Minimal deviation	Limited evidence of terminal metabolic derangement in these two markers. Supports documenting CMSP Core as the primary interpretive summary (cytokine/redox), with vitreous providing minimal additional context.
2-3 (moderate-severe)	0-1 (normal-mild)	Higher glucose *(stress hyperglycemia pattern)*	Compatible with physiologic stress/illness context near death; may be influenced by recent intake/exogenous glucose or resuscitation context when applicable.
0-1 (normal-mild)	2-3 (moderate-severe)	Higher BHB *(ketosis pattern)*	Compatible with reduced intake/fasting interval, intercurrent illness, or catabolic stress physiology. Can support “catabolic context” when corroborated by case history/other markers.
2-3 (moderate-severe)	2-3 (moderate-severe)	Both elevated *(mixed dysregulation pattern)*	Compatible with more marked metabolic dysregulation under severe stress/illness contexts; may support “systemic physiologic disturbance” descriptively when other markers converge.
Any ordinal (0-3)	NS / not scorable	Indeterminate (NS)	None—report NS reason; do not interpret.

^a^ Vitreous glucose and β-hydroxybutyrate (BHB) are reported as contextual physiologic amplifiers only. They do not modify CMSP Core, MVI scoring, archetype assignment, or certification Interpret with standard forensic context, including premortem illness or feeding state, resuscitation or infusion history, and specimen handling/PMI (Appendix C, Section 2.10).

**Table E4:** ** Worked Example of MVI and CMSP Application (Hypothetical Case).** Illustrative stepwise application of normalization, domain scoring, CMSP calculation, and archetype classification within the MVI framework; presented for methodological clarification only.

Domain	Score	Role
1 CYP capacity	3	Core domain
2 Cytokine load	2	Core domain
3 Redox balance	2	Core domain
4 Neurochemical integrity	1	Modifier
5 Xenobiotic/metal burden	2	Modifier/exposure context

**Table F1:** ** Adult Hepatic CYP Protein Abundance Reference Values^a^ (Interpretive Context).** Adult human liver microsomal (HLM) protein abundance plausibility bounds for assay validation and order-of-magnitude checks; not used for scoring.

Isoform	Adult mean ± SD (pmol/mg)	Adult range (min-max) (pmol/mg)	Notes
CYP3A4	85.8 ± 74.6	6.96-246.2	High interindividual variability
CYP2D6	7.90 ± 6.24	0.00-25.1	Strong genotype effect; can be ~0 in poor metabolizers
CYP2C19	5.02 ± 6.68	0.02-25.0	Genotype-dependent; wide spread
CYP3A5	4.00 ± 7.66	0.31-34.2	Expression largely limited to *expressors*; treat as genotype-stratified

**Table F2:** ** CYP3A4 Protein Abundance Age-Matched Developmental Fractions^a^.** Age-specific minimum expected protein levels and suppression tiers expressed as %Adult reference.

Age	Normal (min. expected protein)	Mildly reduced (<20% below min.)	Moderate Suppression (20-30% below min.)	Severe suppression (>30% below min.)
0-7 daysᵃ	0%	--	--	--
1-4 weeks	5%	4%	3%	< 3%
1-2 months	15%	12-14%	11%	< 11%
2-3 months	35%	28-34%	25-27%	< 25%
3-6 months	35%	28-34%	25-27%	< 25%
6-12 months	60%	48-59%	42-47%	< 42%
> 12 months	85%	68-84%	60-67%	<60%

ᵃ Suppression cannot be graded at this age because CYP3A4 protein/capacity is treated as physiologically near zero (consistent with activity assumptions).

**Table F3:** ** CYP2D6 Protein Abundance Age-Matched Developmental Fractions^a^.** Age-specific minimum expected protein levels and suppression tiers expressed as %Adult reference.

Age	Normal (min. expected protein)	Mildly reduced (<20% below min.)	Moderate Suppression (20-30% below min.)	Severe suppression (>30% below min.)
0-7 daysᵃ	0%	--	--	--
1-4 weeks	5%	4%	3%	< 3%
1-2 months	10%	8-9%	7%	< 7%
2-3 months	25%	20-24%	18-19%	< 18%
3-6 months	25%	20-24%	18-19%	< 18%
6-12 months	60%	48-59%	42-47%	< 42%
12 months	85%	68-84%	60-67%	< 60%

ᵃ Suppression cannot be graded at this age because CYP2D6 protein/capacity is treated as physiologically near zero.

**Table F4:** ** CYP2C19 Protein Abundance Age-Matched Developmental Fractions^a^.** Age-specific minimum expected protein levels and suppression tiers expressed as %Adult reference.

Age	Normal (min. expected protein)	Mildly reduced (<20% below min.)	Moderate Suppression (20-30% below min.)	Severe suppression (>30% below min.)
0-4 weeksᵃ	0%	--	--	--
1-2 months	5%	4%	3%	< 3%
2-3 months	20%	16-19%	14-15%	< 14%
3-6 months	20%	16-19%	14-15%	< 14%
6-12 months	60%	48-59%	42-47%	< 42%
12 months	85%	68-84%	60-67%	< 60%

ᵃ Suppression cannot be graded at this age because CYP2C19 protein/capacity is treated as physiologically near zero (consistent with activity assumptions).

**Table F5:** ** CYP3A5 Protein Abundance Age-Matched Developmental Fractions^a^.** Age-specific minimum expected protein levels and suppression tiers expressed as %Adult reference; grading applies to expressors as specified.

Age	Normal (min. expected protein)	Mildly reduced (<20% below min.)	Moderate suppression (20-30% below min.)	Severe suppression (>30% below min.)
0-12 months	70%	56-69%	49-55%	< 49%

^a^ Non-expressers (e.g., CYP3A5 *3/3):* measured protein may be recorded, but suppression is not graded and the isoform score is 0 (normal/physiologic) regardless of the measured value. Values above apply only to CYP3A5 *1 carriers or when genotype is unavailable or indeterminate.

**Table F6:** ** Adult Hepatic CYP Activity Reference Values^a^ (Interpretive Context).** Expected adult probe-substrate activity ranges for assay validation and contextual comparison; not used directly for scoring.

Isoform	Probe Reaction	Expected Adult Range (pmol/min/mg)	Reference Notes
CYP3A4	Midazolam → 1′-hydroxymidazolam	350-450	Consistent with US HLM commercial reference panels; 3-4× variability exists across vendors.
CYP2D6	Dextromethorphan → dextrorphan	120-160	Values represent pooled adult HLM with exclusion of poor-metabolizer phenotypes.
CYP2C19	S-mephenytoin → 4′-hydroxymephenytoin	60-90	Highly genotype-dependent; adult reference pooled across *1/*1 and *1/*2 individuals.
CYP3A5	Midazolam → 1′-hydroxymidazolam	100-250^a^	Activity present only in *CYP3A5* expressors (*1 carriers); adult range must be genotype-stratified.

^a^ CYP3A5 adult ranges vary widely (100-250 pmol/min/mg) among *expressors*. Non-expressors show minimal activity. Laboratories must therefore generate genotype-stratified adult reference values.

**Table F7:** ** CYP3A4 Activity Age-Matched Developmental Fractions^a^.** Age-specific minimum expected activity levels and suppression tiers expressed as %Adult reference.

Age	Normal (min. expected activity)	Mildly reduced (<20% below min.)	Moderate suppression (20-30% below min.)	Severe suppression (>30% below min.)
0-7 days^a^	0%	--	--	--
1-4 weeks	5%	4-4.9%	3.5-3.9%	< 3.5%
1-2 months	20%	16-19%	14-15%	< 14%
2-3 months	30%	24-29%	21-23%	< 21%
3-6 months	40%	32-39%	28-31%	< 28%
6-12 months	50%	40-49%	35-39%	< 35%
> 12 months	80%	64-79%	56-63%	< 56%

**^a^** Suppression cannot be graded at this age because CYP3A4 activity is physiologically near zero.

**Table F8:** ** CYP2D6 Activity Age-Matched Developmental Fractions^a^.** Age-specific minimum expected activity levels and suppression tiers expressed as %Adult reference.

Age	Normal (min. expected activity)	Mildly reduced (<20% below min.)	Moderate suppression (20-30% below min.)	Severe suppression (>30% below min.)
0-7 daysᵃ	0%	--	--	--
1-4 weeks	5%	4-4.9%	3.5-3.9%	< 3.5%
1-2 months	15%	12-14%	10-11%	< 10%
2-3 months	25%	20-24%	17-19%	< 17%
3-6 months	40%	32-39%	28-31%	< 28%
6-12 months	60%	48-59%	42-47%	< 42%
> 12 months	90%	72-89%	63-71%	< 63%

ᵃ Suppression cannot be graded at this age because CYP2D6 activity is physiologically near zero.

**Table F9:** ** CYP2C19 Activity Age-Matched Developmental Fractions^a^.** Age-specific minimum expected activity levels and suppression tiers expressed as %Adult reference.

Age	Normal (min. expected activity)	Mildly reduced (<20% below min.)	Moderate suppression (20-30% below min.)	Severe suppression (>30% below min.)
0-7 daysᵃ	0%	--	--	--
1-4 weeks	10%	8-9%	7%	< 7%
1-2 months	25%	20-24%	18-19%	< 18%
2-3 months	35%	28-34%	25-27%	< 25%
3-6 months	50%	40-49%	35-39%	< 35%
6-12 months	70%	56-69%	49-55%	< 49%
> 12 months	90%	72-89%	63-71%	< 63%

ᵃ Suppression cannot be graded at this age because CYP2C19 activity is physiologically near zero.

**Table F10:** ** CYP3A5 Activity Age-Matched Developmental Fractions^a^.** Age-specific minimum expected activity levels and suppression tiers expressed as %Adult reference; grading applies to expressors as specified.

Age	Normal (min expected activity)	Mildly reduced (<20% below min.)	Moderate suppression (20-30% below min.)	Severe suppression (>30% below min.)
0-7 days	10%	8-9%	7%	< 7%
1-4 weeks	20%	16-19%	14-15%	< 14%
1-2 months	30%	24-29%	21-23%	< 21%
2-3 months	40%	32-39%	28-31%	< 28%
3-6 months	50%	40-49%	35-39%	< 35%
6-12 months	70%	56-69%	49-55%	< 49%
> 12 months	90%	72-89%	63-71%	< 63%

^a^ Suppression cannot be graded for non-expressors whose activity is physiologically minimal. Values apply only to CYP3A5 *1 carriers or when genotype is unavailable or indeterminate.

**Table G1:** ** Summary of Operational Roles and Responsibilities.** Professional roles, primary responsibilities, and associated analytic platforms supporting implementation of workflows described in Appendices C-E.

Professional Role	Primary Responsibilities	Relevant Sections (C-E)	Core Equipment / Platforms
1.1 Forensic Pathologist	Oversees autopsy; performs gross exam; selects tissue blocks; orders ancillary studies; integrates findings	C: 1-2, 2.5, 2.9-2.10, 4-5 E: 1-3	Standard autopsy suite; microtome; histology workflow; documentation system
1.2 Pediatric Neuropathologist	Brain dissection; region-specific sampling; IHC interpretation; correlates structural and biochemical injury	C: 2.1, 2.5, 4-5 D: 4 E: 2.5, 5, 7	Formalin fixation; paraffin embedding; IHC staining systems; microscopy
1.3 Forensic Histotechnologist	Tissue processing; embedding; cutting; staining (H&E and targeted IHC panels)	C: 2.1, 2.5 D: 4 E: 2.4	Microtome; automated stainers; antigen retrieval systems; slide scanners
1.4 Clinical Toxicologist	Screens for xenobiotic/metals (Domain 5 burden), aldehydes, excipients, metabolites	C: 2.3-2.5, 2.9 D: 5 E: 2.5	GC-MS, LC-MS/MS, ICP-MS; derivatization kits
1.5 Mass Spectrometry Technologist	Performs CYP450 activity assays, neurochemical quantification, mitochondrial ATP quantification	C: 2.1, 2.5, 2.9-2.10 D: 1,3 E: 2.1, 2.3	LC-MS/MS; GC-MS; HPLC-ECD; LC-ECD; Oroboros O2k
1.6 Molecular Diagnostics Specialist	Performs pharmacogenetic testing; evaluates CYP2D6, CYP2C19, CYP3A4/5 variants	C: 4 (conditional triggers); D: 1.4, 4.2 E: 2.1, 2.4, 3.2	PCR systems; Sanger sequencing; NGS panels; allele-calling software
1.7 Clinical-Immunologist / Cytokine Lab	Quantifies IL-6, IL-1β, TNF-α, CRP, and contextual immune markers (e.g., CCL2/MCP-1)	C: 2.1; 2.9-2.10 D: 2; E: 2.2, 4.1	ELISA; multiplex bead arrays; nephelometry
1.8 Redox/ Mitochondrial Biochemist	Measures glutathione ratios; oxidative markers (8-OHdG, MDA); ATP depletion	C: 2.1, 2.9; D: 3.4 E: 4.1-4.2, 7	LC-ECD; HPLC; spectrophotometry; ATP quantification assays
1.9 Electron Microscopy Technologist	TEM ultrastructural assessment of mitochondria, capillaries, tight junctions	C: 2.3-2.5, 2.9 D: 5.3; E: 2.5	TEM; ultramicrotome; heavy-metal staining
1.10 Data Scientist/ LIMS Manager	Implements data capture; computes MVI domain and scores per Appendix E (no modification of thresholds or interpretive rules)	C: 2.9-2.10, 5 E: 2-5, 7	Laboratory Information Management System (LIMS); dashboarding tools
1.11 Quality Assurance Officer	Oversees assay validity; ensures compliance with CAP/ISO standards; repeats outlier analyses	C 2.7; D 2; D 4	QA/QC SOP; calibration records; proficiency testing datasets

**Table H1:** ** Single-Domain MVI Reference Archetypes^a^ (Ref. #1-5).** Standardized mechanistic interpretations for patterns in which a single MVI domain has a non-zero score.

*Ref. No. * *Domain Pattern* *(Mechanistic Class)*	*Mechanism*
***#1** Isolated CYP impairment* *(Isolated metabolic limitation)*	Reduced hepatic CYP450 metabolic capacity (enzyme activity below age-matched developmental expectations after adult normalization) without evidence of cytokine-mediated phenoconversion, redox/energetic failure, neurochemical instability, or xenobiotic/metal overload. Most consistent with physiologic immaturity or genotype-limited reserve; does not indicate multi-axis metabolic failure or terminal collapse.
***#2** Isolated Cytokine Elevation* *(Isolated immune activation)*	*Systemic inflammatory activation (cytokines elevated relative to assay- and age-specific reference ranges) without reduced CYP capacity consistent with phenoconversion, redox/energetic failure, neurochemical instability, or xenobiotic/metal overload. Consistent with early or isolated immune signaling; does not demonstrate phenoconversion or convergent mechanisms sufficient to explain collapse in isolation.*
***#3** Isolated Redox Depletion* *(Isolated oxidative/ mitochondrial stress)*	*Oxidative-mitochondrial stress (redox imbalance and/or reduced energetic reserve) with preserved CYP450 capacity and no marked immune activation or xenobiotic/metal overload. Consistent with hypoxic exposure, mitochondrial vulnerability, or intrinsic metabolic disease; does not support cytokine-mediated suppression or multi-axis collapse by itself.*
***#4** Isolated Neurochemical Imbalance* *(Isolated autonomic vulnerability)*	Neurochemical dysregulation (e.g., serotonergic or neurosteroid pattern shifts) without systemic immune activation, redox depletion, impaired CYP capacity, or xenobiotic/metal overload. Supports intrinsic autonomic/arousal vulnerability; may indicate susceptibility to dysregulated respiratory or autonomic control without evidence of metabolic collapse.
***#5** Isolated Xenobiotic/metal Burden* *(Exposure without overload)*	Xenobiotic, excipient, or metal exposure above age-referenced background with preserved CYP450 capacity, no marked immune activation, intact redox balance, and stable neurochemical indices. Indicates exposure without evidence of overload, impaired clearance, or toxicodynamic failure; does not support metabolic collapse as a primary mechanism.

**Table H2:** ** Two-Domain Convergence MVI Reference Archetypes^a^ (Ref. #6-10).** Standardized interpretations for interacting two-domain configurations, including defined referral rules to avoid overlapping archetype descriptions.

*Ref. No.* *Domain Pattern* (Mechanistic Class)	*Mechanism*
#6 CYP + Cytokine (Immune-mediated phenoconversion)	Integrated findings show inflammatory activation with reduced CYP450 metabolic capacity relative to age-matched expectations, consistent with cytokine-mediated phenoconversion. Redox failure and xenobiotic/metal overload are not required for this pattern. This configuration reflects reduced functional clearance capacity under active immune signaling.
#7 CYP + Redox (Metabolic-mitochondrial coupling failure)	Findings show concurrent reduction in CYP450 metabolic capacity and oxidative-mitochondrial stress without significant inflammatory activation. This convergence indicates impaired metabolic efficiency with reduced redox/energetic reserve, lowering physiologic resilience without meeting criteria for multi-axis collapse.
#8 Cytokine + Redox (Inflammatory-oxidative amplification)	Findings demonstrate inflammatory activation with oxidative-mitochondrial stress while CYP450 capacity is relatively preserved. This pattern reflects inflammatory-oxidative amplification in which immune signaling and redox depletion jointly destabilize metabolic homeostasis.
#9 Cytokine or Redox + Neurochemical (Autonomic destabilization under systemic stress)	Neurochemical instability occurs in conjunction with either inflammatory activation or oxidative-mitochondrial stress. This pattern reflects autonomic/arousal dysregulation under systemic physiologic strain and may impair adaptive responses (e.g., to hypoxia or infection) without primary CYP suppression.
#10 CYP or Redox + Xenobiotic (Exogenous load exceeding reserve)	Xenobiotic, excipient, and/or metal burden is present in the context of reduced CYP450 capacity and/or oxidative-mitochondrial stress, indicating exogenous load in a system with constrained clearance and/or redox buffering. This reflects interaction between exposure and vulnerability rather than isolated toxicity.
# —(Referral) CYP + Neurochemical	Assign Ref #1 (Isolated CYP impairment). Document Neurochemical as modifier (Domain 4).
# —(Referral) Cytokine + Xenobiotic/metal	Assign Ref #2 (Isolated Cytokine elevation)/ Document Xenobiotic/metal as modifier (Domain 5).
# —(Referral) Neurochemical + Xenobiotic	Assign Ref #4 if Neurochemical is dominant; assign Ref #5 if Xenobiotic is dominant; document the other domain as a modifier.

^a^ Two-domain archetypes are labeled by interacting domains only; qualifiers are confined to definitions to avoid presuming severity or outcome.

**Table H3:** ** Three-Domain Convergence MVI Reference Archetypes (Ref. #11-13).** Core multi-domain interaction patterns with defined referral logic for modifier domains.

*Ref. No.* *Domain Pattern* (Mechanistic Class)	*Mechanism*
#11 CYP + Cytokine + Redox (Core-Multi-Axis Metabolic Collapse)	Findings show reduced CYP450 metabolic capacity with active inflammatory signaling and oxidative-mitochondrial depletion. The combined pattern indicates constrained reserve across clearance, immune modulation, and redox/energetic buffering. This is the canonical CYP-cytokine-redox convergence pattern in the MVI.
# —(Referral) CYP + Cytokine + Neurochemical	Assign Ref #6 (CYP + Cytokine; Immune-mediated phenoconversion) as the core pattern. Document Neurochemical as a modifier (Domain 4).
#12 Cytokine + Redox + Neurochemical (Inflammatory-oxidative autonomic failure)	Findings show inflammatory activation with oxidative-mitochondrial stress and neurochemical dysregulation, with relatively preserved CYP450 capacity. This pattern supports inflammatory-oxidative destabilization of autonomic/arousal regulation without primary CYP suppression.
# —(Referral) Cytokine + Redox + Xenobiotic/metal	Assign Ref #8 (Cytokine + Redox; Inflammatory-oxidative amplification) as the core pattern. Document Xenobiotic/metal as exposure context (Domain 5).
#13 CYP + Redox + Neurochemical (Neuro-metabolic vulnerability)	Findings show reduced CYP450 capacity with oxidative-mitochondrial depletion and neurochemical dysregulation. Constrained clearance together with reduced redox/energetic reserve may contribute to downstream serotonergic or neurosteroid pattern shifts, increasing autonomic/arousal vulnerability. This pattern reflects a neuro-metabolic vulnerability state in which impaired clearance and redox buffering coincide with neurochemical dysregulation.
# —(Referral) CYP + Neurochemical + Xenobiotic/metal	Assign Ref #10 (CYP or Redox + Xenobiotic; Exogenous load exceeding reserve) as the core pattern. Document Neurochemical (Domain 4) as a modifier.
# —(Referral) Cytokine + Neurochemical + Xenobiotic/metal	Assign Ref #2 (Isolated Cytokine elevation) as the core pattern. Document Xenobiotic/metal (Domain 5) as exposure context and Neurochemical (Domain 4) as a modifier. If Domain 5 score > Domain 2 score, assign Ref #5 (Isolated Xenobiotic/metal burden) instead and document Domain 2 and Domain 4 as modifiers.
# —(Referral) Redox + Neurochemical + Xenobiotic/metal	Assign Ref #10 (CYP or Redox + Xenobiotic; Exogenous load exceeding reserve) as the core pattern. Document Neurochemical (Domain 4) as a modifier.
#14 CYP + Cytokine or Redox + Xenobiotic/metal (Terminal Multi-Axis Overload)	Findings show xenobiotic, excipient, or metal burden above age-referenced background in the context of reduced CYP450 capacity and inflammatory signaling and/or oxidative-mitochondrial stress. This pattern reflects multi-axis overload in which exogenous burden coincides with constrained clearance and buffering capacity. It supports interpretation of combined exposure and physiologic susceptibility rather than isolated toxicity.

**Table H4:** ** Four- or Five-Domain Convergence MVI Reference Archetypes.** Referral-based framework for complex multi-domain configurations assigning a core archetype with documented modifier or exposure domains.

*Ref. No.* Domain Pattern	*Mechanism*
# —(Referral) CYP + Cytokine + Redox + Neurochemical or Xenobiotic	Assign Ref #11 (CYP + Cytokine + Redox; Core-Multi-Axis Metabolic Collapse as the core pattern. Document the appropriate modifier—either Neurochemical (Domain 4) or Xenobiotic/metal (Domain 5). Domain 5 may indicate concurrent overload pressure.
# —(Referral) CYP + Cytokine + Neurochemical + Xenobiotic	Assign Ref #14 (CYP + Cytokine or Redox + Xenobiotic/metal; Terminal Multi-Axis Overload) as the core pattern via CYP + Cytokine + Xenobiotic/metal. Document Neurochemical (Domain 4) as a modifier.
# —(Referral) CYP + Redox + Neurochemical + Xenobiotic	Assign Ref #14 as the core pattern via CYP + Redox + Xenobiotic/metal. Document Neurochemical (Domain 4) as a modifier.
# —(Referral) Cytokine + Redox + Neurochemical + Xenobiotic	Assign Ref #8 (Cytokine + Redox; Inflammatory-oxidative amplification) as the core pattern. Document Neurochemical (Domain 4) as a modifier and Xenobiotic/metal (Domain 5) as exposure context/modifier.
# —(Referral) CYP + Cytokine + Redox + Neurochemical + Xenobiotic	Assign Ref #11 (Core-Multi-Axis Metabolic Collapse) as the core pattern. Document Neurochemical (Domain 4) as a modifier and Xenobiotic/metal (Domain 5) as exposure context/modifier.

**Table I1:** ** Design Rationale and Interpretive Safeguards of the MVI.** Summary of core design principles, methodological safeguards, and scope limitations underlying the MVI framework.

Question / Concern	Design Principle	Framework Rationale and Response
Why was the MVI developed?	Operationalization of theory	The MVI translates the Three-Axis Convergence Framework (TCF) into a structured, reproducible analytic tool suitable for postmortem evaluation and mechanistic pattern recognition.
Why these five domains?	Mechanistic distinctness	The five MVI domains—CYP450 capacity, cytokine load, redox balance, neurochemical integrity, and xenobiotic/metal burden—represent distinct physiologic constraints on metabolic reserve, each grounded in independent literatures.
Are domains redundant or overlapping?	Functional non-redundancy	Domains were selected to represent independent failure modes (enzymatic capacity, immune suppression, energetic stress, autonomic regulation, and exposure burden) rather than correlated biomarkers, minimizing redundancy while allowing structured convergence analysis.
Why use an ordinal 0-3 scoring system?	Avoidance of false precision	A coarse ordinal 0-3 scale captures biologically meaningful severity tiers while avoiding false precision inherent in continuous scoring under postmortem degradation, biologic variability, and analytic uncertainty.
Why are the CYP450 thresholds set at 75/50/25%?	Functional reserve stratification	These thresholds reflect stepwise reductions in metabolic reserve relative to age-matched expectation, distinguishing preserved reserve, partial compromise, marked impairment, and critical limitation without assuming linear dose-response relationships.
Why is cytokine load scored by fold elevation?	Assay- and age-normalized comparability	Fold-based scoring ensures comparability across assays and developmental stages, capturing biologically meaningful inflammatory amplification while avoiding misclassification due to platform- or age-dependent variability in absolute concentrations.
Is the scoring arbitrary?	Constrained, domain-specific criteria	Scoring is applied only where reduced biologic capacity or burden can be inferred from operationally robust measures anchored to reference expectations (e.g., hepatic CYP protein abundance; tissue F2-isoprostanes). Ordinal tiers are used to avoid overfitting, additive inflation, or unsupported precision.
How is developmental immaturity distinguished from suppression?	Two-step normalization	CYP450 capacity is interpreted by normalizing measured protein abundance (or QC-qualified activity) to an adult reference and then comparing to age-matched developmental expectations, preventing physiologic immaturity from being misclassified as pathologic suppression.
Is the MVI diagnostic or causal?	Interpretive limitation	The MVI is not a diagnostic instrument and does not assign cause of death; it supports standardized mechanistic interpretation within established forensic frameworks.

**Table J1:** ** Glossary of Abbreviations and Analytic Terms.** Definitions of abbreviations and technical terms used throughout the MVI framework and appendices.

Abbreviation	Meaning
5-HIAA	5-Hydroxyindoleacetic acid; primary serotonin (5-HT) metabolite used to assess serotonergic turnover.
5-HT	Serotonin; neurotransmitter involved in arousal, autonomic regulation, and respiratory control.
8-OHdG	8-Hydroxy-2′-deoxyguanosine; biomarker of oxidative DNA damage.
ATP	Adenosine triphosphate; indicator of cellular and mitochondrial energy status.
BHB	β-hydroxybutyrate; a ketone body produced during fasting or metabolic stress, serving as a marker of systemic energy balance and catabolism.
CAP	College of American Pathologists; organization providing laboratory accreditation and proficiency testing standards.
CCL2 (MCP-1)	C-C motif chemokine ligand 2; marker of macrophage activation and inflammatory particle migration.
CMSP	Cytokine-Metabolic Suppression Profile; composite metric integrating cytokine load, CYP activity, redox status, and endocrine stress.
CRP	C-reactive protein; nonspecific acute-phase marker of systemic inflammation.
CSF	Cerebrospinal fluid.
CYP	Cytochrome P450 enzyme family responsible for Phase I metabolic clearance.
DNPH	2,4-Dinitrophenylhydrazine; a chemical reagent commonly used to detect aldehydes and ketones by forming a visible precipitate.
EDTA	Ethylenediaminetetraacetic acid; anticoagulant used in blood collection.
ELISA	Enzyme-linked immunosorbent assay for quantitative measurement of proteins, cytokines, or hormones.
EM	Electron microscopy; ultrastructural imaging technique for cellular, mitochondrial, and microvascular assessment.
F2-isoprostanes	Stable lipid peroxidation products formed by free-radical oxidation of arachidonic acid and widely used as quantitative biomarkers of oxidative stress.
GC-MS	Gas chromatography-Mass Spectrometry; an analytical technique that separates chemical compounds and identifies them based on mass spectra.
H&E	Hematoxylin and eosin stain; routine histologic stain.
HLM	Human liver microsomes; standardized preparation used in CYP450 activity assays.
HPLC	High-performance liquid chromatography; analytical separation technique.
HPLC-ECD	HPLC with electrochemical detection; used for neurotransmitters and redox markers.
ICP-MS	Inductively coupled plasma mass spectrometry; sensitive quantification of metals.
IHC	Immunohistochemistry; tissue-based protein localization technique.
IL-1β	Interleukin-1 beta; pro-inflammatory cytokine and upstream immune amplifier.
IL-6	Interleukin-6; cytokine associated with suppression of CYP450 transcription.
iso-PGF2α	8-iso-prostaglandin F2α: A stable F2-isoprostane used as a validated LC-MS/MS biomarker of oxidative stress and redox imbalance.
LC-ECD	Liquid chromatography with electrochemical detection; used for oxidative and redox markers.
LC-MS/MS	Liquid chromatography-tandem mass spectrometry; reference platform for quantifying metabolites, xenobiotics, and CYP activity.
LIMS	Laboratory information management system; digital infrastructure for lab data handling.
MDA	Malondialdehyde; lipid peroxidation marker.
MVI	Metabolic Vulnerability Index; composite scoring system across metabolic, immune, redox, neurochemical, and xenobiotic domains.
N₂	Liquid nitrogen; used for rapid tissue freezing.
NGS panel	Next-generation sequencing panel for pharmacogenetic allele detection.
NS	Not scorable—analyte/result does not meet prespecified specimen validity criteria.
Oroboros O2k	High-resolution oxygraph for mitochondrial oxygen-consumption measurements.
PCR	Polymerase chain reaction; DNA amplification technique.
PGx	Pharmacogenomics; the use of genetic information to predict drug response.
PMI	Postmortem Interval—the time between death and specimen collection.
QA	Quality assurance; systematic processes ensuring laboratory compliance.
QC	Quality control; procedures monitoring analytical accuracy and precision.
SERT (5-HTT)	Serotonin transporter; regulates presynaptic serotonin reuptake.
SIDS	Sudden Infant Death Syndrome; unexplained death of an infant under one year.
SOP	Standard Operating Procedure—a written protocol outlining a procedure.
SSRI	Selective serotonin reuptake inhibitor; class of antidepressant drugs.
TEM	Transmission electron microscopy; ultrastructural imaging technique.
TNF-α	Tumor necrosis factor-alpha; pro-inflammatory cytokine with CYP-suppressive effects.
TPH2	Tryptophan hydroxylase 2; rate-limiting enzyme in central serotonin synthesis.

## Abbreviations

ADH5: Alcohol dehydrogenase 5
ALDH: Aldehyde dehydrogenase
ATSDR: Agency for Toxic Substances and Disease Registry
CO₂: Carbon dioxide
CRP: C-reactive protein
CYP or CYP450: Cytochrome P450 enzyme superfamily
DTaP: Diphtheria, tetanus, acellular pertussis vaccine
EFSA: European Food Safety Authority
FDA: U.S. Food and Drug Administration
HepA: Hepatitis A vaccine
HepB: Hepatitis B vaccine
IL-1β: Interleukin-1 beta
IL-6: Interleukin-6
IPV: Inactivated poliovirus vaccine
MMR: Measles, mumps, rubella vaccine
MVI: Metabolic Vulnerability Index
NADPH: nicotinamide adenine dinucleotide phosphate
PCV13: 13-valent pneumococcal conjugate vaccine
PK: Pharmacokinetics
RCT: Randomized controlled trial
SBS: Shaken Baby Syndrome
SIDS: Sudden Infant Death Syndrome
SSRI: Selective Serotonin Reuptake Inhibitor
SUID: Sudden Unexpected Infant Death
TCF: Three-Axis Convergence Framework
TNF-α: Tumor necrosis factor alpha

## Appendix A. The CYP450 Catalytic Cycle and Monooxygenase Function

Cytochrome P450 (CYP450) enzymes mediate primary Phase I oxidative biotransformation (detoxification and, in some contexts, bioactivation). As heme-thiolate monooxygenases, they insert one atom of molecular oxygen (O₂) into a substrate while reducing the second to water (H₂O), increasing polarity and preparing products for Phase II conjugation and elimination. In early life, the liver is substantially smaller (often cited as ~one-tenth adult scale) and hepatic CYP expression is developmentally immature, making overall clearance capacity highly dependent on isoform ontogeny, oxygen delivery, and NADPH availability. Even at this reduced early-life scale, the liver's CYP system executes a massive throughput—millions of enzyme-catalyzed oxidation events per second across the organ.

The catalytic cycle can be summarized in six recurring steps:

1. Binding: A substrate (the molecule being metabolized) enters the active site, displacing bound water and shifting the heme iron into an O₂-reactive state.

2. O₂ binding: Molecular oxygen (O₂) binds the heme iron—this is for chemical activation, not oxygen storage (unlike hemoglobin).

3. Electron delivery: NADPH supplies electrons via cytochrome P450 reductase; in some reactions, cytochrome b₅ serves as an auxiliary electron donor/modulator, reducing the heme-O₂ complex.

4. Activation: The O₂ bond is cleaved to form a highly reactive iron-oxo (“activated oxygen”) intermediate; the second oxygen atom is reduced to H₂O.

5. Insertion: The activated oxygen is inserted into the substrate—often as hydroxylation (R-H → R-OH, where R denotes the substrate's carbon framework/“rest of the molecule”)—increasing polarity and enabling Phase II conjugation.

6. Release: The oxidized product dissociates; it may then undergo Phase II conjugation (e.g., glucuronidation/sulfation) followed by transport and excretion.

### When CYP450 Metabolic Capacity Is Exceeded

Clinical consequences may include prolonged drug half-life, increased toxicity at standard doses, and heightened sensitivity to drug-drug interactions. Mechanistically, substrates accumulate in plasma and tissues, and excess substrate may be diverted into alternative metabolic pathways that generate reactive intermediates. Cofactors, including NADPH and electron-transfer proteins, can become locally depleted, promoting oxidative stress and endoplasmic reticulum strain and impairing hepatocyte function.

### Biological Significance

The CYP450 catalytic cycle operates continuously in hepatocytes and depends on adequate oxygen supply, NADPH availability, heme integrity, redox balance, and inflammatory status. These dependencies make CYP450 activity highly sensitive to metabolic stress—particularly in early infancy, when redox systems, immune regulation, and enzyme ontogeny are still developing. This sensitivity aligns with the Three-Axis Convergence Framework (TCF), which highlights how developmental immaturity, cytokine activation, and cumulative xenobiotic exposure may converge to create periods of heightened metabolic vulnerability.

## Appendix B. Mechanistic Case Examples of the Three-Axis Convergence Framework

The following examples illustrate how variation within the Three-Axis Convergence Framework (TCF) can constrain metabolic reserve across different life stages. These scenarios are not intended to establish causation or predict outcomes, but to demonstrate biologically grounded interactions among CYP450 capacity, inflammatory signaling, and xenobiotic or excipient burden as they manifest in real-world pharmacokinetic contexts.

Within the TCF, each axis independently constrains metabolic reserve:

• **Axis 1:** developmental immaturity or genetically reduced CYP450 metabolic capacity

• **Axis 2:** cytokine-mediated suppression of CYP activity (phenoconversion)

• **Axis 3:** cumulative xenobiotic or excipient burden

Neurochemical disturbances (e.g., serotonergic or neurosteroid imbalance) are treated as downstream functional correlates of metabolic or inflammatory constraint rather than independent primary axes. Redox and mitochondrial stress are treated as physiologic amplifiers that lower the threshold at which autonomic dysregulation and clinical instability emerge.

Although each axis may contribute to vulnerability on its own, overlapping influences can further constrain metabolic clearance during critical developmental or physiologic states.

### Example 1 — Infant: Axis 1 (immaturity) + Axis 2 (inflammation)

A 10-week-old infant, within a developmental window of markedly reduced CYP3A4/5 capacity and carrying a CYP3A5 non-expressor genotype, is hospitalized with a severe bacterial lower-respiratory infection. The illness triggers an IL-6-dominant inflammatory response that suppresses CYP3A expression (Axis 2). During respiratory support in the intensive care unit, the infant receives midazolam, a sedative primarily cleared by CYP3A4/5. Because inflammation further reduces CYP3A activity, midazolam is eliminated more slowly than expected, increasing the risk of accumulation and prolonged or deep sedation even at standard infusion rates.

This example illustrates how cytokine-driven phenoconversion (Axis 2) can further constrain an already limited metabolic reserve in early infancy due to developmental and genetic factors (Axis 1).

### Example 2 — Adult: Axis 1 (genetic constraint) + Axis 3 (inhibitor load)

An adult male with a CYP2D6 intermediate-metabolizer genotype has reduced baseline capacity to clear CYP2D6 substrates (Axis 1). He takes metoprolol for hypertension. After developing dermatitis, he begins using diphenhydramine (Benadryl™), a moderate CYP2D6 inhibitor (Axis 3). As CYP2D6 activity decreases further, metoprolol clearance declines, increasing circulating levels and producing dizziness, fatigue, or lightheadedness despite an unchanged dose.

This example demonstrates how modest inhibitor exposure (Axis 3) can amplify genetic metabolic constraints (Axis 1), producing clinically meaningful shifts in pharmacokinetics. The resulting neurobehavioral effects reflect downstream neurotransmitter overstimulation due to impaired metabolic clearance, rather than a primary neurochemical vulnerability independent of metabolic constraint.

### Example 3 — Psychotropic Exposure: Axis 1 (genotype-driven metabolic constraint)

A young adult with a CYP2D6 poor-metabolizer genotype begins treatment with paroxetine, a serotonin-modulating SSRI predominantly cleared by CYP2D6 and also acting as a CYP2D6 inhibitor. Because the individual's metabolic capacity is markedly reduced at baseline (Axis 1), paroxetine accumulates even at standard doses, and the drug's auto-inhibitory effects further elevate serum concentrations. Within days, the individual develops severe akathisia, intense dysphoria, emotional agitation, and intrusive violent impulses—symptoms that may be misinterpreted as worsening psychiatric illness rather than toxicodynamic overstimulation.

This example shows how Axis 1 constraints alone, when combined with reliance on an impaired metabolic pathway, can precipitate profound neurobehavioral instability in a susceptible individual. It illustrates toxicodynamic amplification driven by metabolic constraint rather than primary psychiatric pathology.

### Contextual Orientation and Methods Overview (Cross-Appendix)

The mechanistic examples in this appendix illustrate core principles of the Three-Axis Convergence Framework and are provided for conceptual orientation rather than evidentiary inference. Implementation of this framework follows a structured postmortem workflow that integrates standardized case review, specimen collection, and conditional testing across the five physiologic domains of metabolic vulnerability. Operational procedures for specimen handling and prioritization are defined in Appendix C; quantitative analytic methods and quality-control conventions for CYP450 capacity, xenobiotic and metal burden, cytokine and immune activation, redox/oxidative-stress anchors, and neurochemical integrity are specified in Appendix D. These domain-level findings are synthesized using the Metabolic Vulnerability Index (MVI) and CMSP scoring framework defined in Appendix E, which applies age-adjusted thresholds to generate an ordinal vulnerability profile without assigning cause of death. Reference archetypes, interpretive safeguards, and design rationale are provided in Appendices H and I, respectively, with CYP protein abundance and activity reference tables provided in Appendix F. Operational roles and responsibilities for implementation across Appendices C-E are mapped in Appendix G. Abbreviations and defined terms are provided in the Glossary (Appendix J).

## Appendix C. Operational Postmortem Workflow, Specimen Collection, and Conditional Testing Framework

### Scope and boundary

This appendix defines the operational workflow for postmortem case review, specimen collection, handling, prioritization, and conditional testing in unexplained infant deaths. It does not define analytic methods, mechanistic interpretation, or MVI/CMSP scoring beyond the operational requirements for specimen validity. Detailed analytic methods and QC conventions are provided in Appendix D; domain scoring and worked examples are provided in Appendix E.

Cause-of-death certification follows established forensic standards. The Metabolic Vulnerability Index (MVI) and Cytokine-Metabolic Suppression Profile (CMSP) operate within this framework and do not replace it.

Operational activities are organized according to the five MVI domains used for scoring: Domain 1 CYP450 capacity; Domain 2 Cytokine load; Domain 3 Redox balance; Domain 4 Neurochemical integrity; Domain 5 Xenobiotic/metal burden.

### Decision framework for cause-of-death certification

**Step 1: Proximate cause.** If a proximate cause is identified (e.g., mechanical asphyxia, infection, congenital disease, trauma, poisoning), certify accordingly.

**Step 2: Biologic vulnerability/mechanism.** If no proximate cause is identified, evaluate for demonstrable biologic mechanism or vulnerability (e.g., channelopathies/epilepsy, inborn errors, cardiomyopathy, convergent metabolic vulnerability). If vulnerability is identified without a proximate cause, certify **Undetermined** with documented contributing mechanism; cases may still qualify for SUID surveillance per jurisdiction.

**Step 3: Residual classification.** SIDS is applied only when no proximate cause and no biologic mechanism is demonstrable after complete investigation, in an infant <1 year, consistent with diagnosis-of-exclusion conventions.

### Boundary statement

The MVI characterizes physiologic vulnerability patterns that may limit tolerance to stressors; it does not assign cause of death or establish lethality. MVI/CMSP findings may support mechanistic documentation in cases certified *Undetermined,* but do not substitute for standard forensic criteria or the diagnosis-of-exclusion framework for SIDS.

### 1. Pre-analytic case review (contextual only)

1.1 Document available clinical and environmental history (no presumption of causality), including:

- recent illness/fever and recent routine immunizations (recorded for temporal context only; no causal inference)
- medications or excipient exposures (e.g., ethanol-, polysorbate-containing products)
- gestational age, growth parameters, prior apneic events, congenital anomalies

1.2 Obtain maternal history, including pharmacogenetic data if available (CYP2D6, CYP2C19, CYP3A4/5, CYP1A2).

1.3 Record environmental stressors (sleep position, temperature, potential toxin exposure, hypoxic settings).

### 2. Specimen collection, handling, and prioritization

#### 2.0 Operational target

Target collection and preservation within **≤24 hours PMI** when feasible. Collection remains informative beyond this window when cold chain is verified and the specimen falls within the stability windows in Section 2.9.

#### 2.1 Primary specimen set (recommended)

##### Liver (frozen; standardized lobe when feasible)

- Purpose: Domain 1 CYP450 capacity (protein abundance anchor; activity conditional/QC-qualified only), Domain 3 redox anchor (tissue F2-isoprostanes), Domain 5 xenobiotic/metal (tissue tox confirmation and/or ICP-MS as indicated).

##### Brainstem (medulla ±pons; FFPE required; frozen optional)

- Purpose: Domain 4 neuropathologic/serotonergic integrity anchors (H&E + IHC). Frozen tissue (if available) supports optional contextual neurochemistry (e.g., 5-HT/5-HIAA).

##### Femoral venous blood (not cardiac; serum preferred; EDTA plasma acceptable)

- Purpose: Domain 2 cytokine anchors (IL-6, CRP), toxicology (primary matrix when available).

##### Vitreous humor (both eyes per SOP; record pooled vs separate)

- Purpose: contextual physiologic amplifier (non-scoring for MVI/CMSP Core); vitreous glucose + β-hydroxybutyrate.

##### Kidney cortex (optional corroboration; frozen)

- Purpose: corroborative redox marker when liver is unavailable/compromised; optional metals/toxicology corroboration.

**Note on urine/bile:** Urine is frequently unavailable or insufficient in infant postmortem examinations. Urine-dependent oxidative markers are not required for CMSP scoring.

#### 2.2 Optional immune-organ sampling (contextual)

When routinely obtained in SUID/SIDS autopsy, retain lung, spleen, and thymus for histology; retain CSF when available for contextual neuroimmune assessment. These specimens are contextual only and are not required for MVI domain scoring.

#### 2.3 Xenobiotic/metal sampling (routine operational minimum)

To support Domain 5 scoring, obtain specimen(s) sufficient for comprehensive toxicology and metals. Preferred matrices are jurisdiction- and laboratory-dependent; at minimum retain:

- **Blood** for comprehensive toxicology when available (preferred: peripheral/femoral).
- **Liver (frozen)** for tissue toxicology confirmatory testing and **metals (ICP-MS)** when metals burden is assessed.
- **Bone (optional)** for confirmatory metals when clinically/forensically indicated.

#### 2.4 Chain-of-custody

Maintain continuous chain-of-custody documentation for all biospecimens.

#### 2.5 Specimen prioritization when tissue is limited

If partial autopsy, decomposition, or limited tissue availability:

1. **Liver (≥0.3 g; frozen):** Domain 1 (CYP protein abundance), Domain 3 (tissue F2-isoprostanes), Domain 5 (metals/toxicology corroboration as indicated).

1. **Brainstem (medulla; FFPE minimum; ± frozen if feasible):** Domain 4 anchors (H&E + serotonergic IHC).

3. **Femoral blood:** Domain 2 anchors (IL-6, CRP) + toxicology.

4. **Vitreous:** glucose + β-hydroxybutyrate (contextual amplifier).

**5. Kidney cortex (frozen):** Domain 3 corroboration when liver is compromised or unavailable; optional Domain 5 corroboration.

**Minimum recommended set to enable 5-domain MVI scoring:** liver (frozen), brainstem (FFPE), blood (serum/plasma), and specimen(s) adequate for comprehensive toxicology ± metals; vitreous is recommended for contextual physiologic amplification.

#### 2.9 Approximate postmortem stability windows (guidance)

##### Post-collection processing target (applies unless overridden by assay SOP)

Blood—centrifuge/separate serum or plasma within 2 hours of specimen collection at autopsy (not time of death) and refrigerate immediately (freeze at -80°C preferred).

Tissue—collect early and snap-freeze within 30 minutes of sampling (-80°C preferred; avoid repeat freeze-thaw).

Vitreous—refrigerate immediately and freeze if analysis is delayed; record whether left/right eyes were analyzed separately or pooled.

Indicative ranges assume prompt refrigeration and rapid freezing; practical targets rather than validated cutoffs.

#### 2.10 CMSP analyte validity and scorable/NS criteria (operational)

A CMSP biomarker result is scorable only if:

- The correct specimen/matrix was obtained (e.g., femoral venous blood, not cardiac blood),
- The postmortem interval (PMI) is within the “Best within / Max PMI (sampling)” guidance window (Table 2.9), and
- post-collection processing targets were met (Section 2.9; timing referenced to specimen collection at autopsy, not time of death) with documented cold chain.

If any condition is not met, mark the analyte NS (not scorable) for CMSP purposes (numeric result may still be reported descriptively with the relevant pre-analytic limitations documented).

### 3. Analytic domains (procedural context only)

Routine postmortem analyses may proceed across the five operational domains based on specimen availability and case context. Appendix C defines operational sequencing and documentation; analytic methods, QC conventions, and interpretive constraints are provided in Appendix D; MVI/CMSP scoring is provided in Appendix E.

### 4. Operational triggers for conditional testing (realistic postmortem set)

Conditional studies may be triggered by abnormal routine findings within the same stream. This section specifies operational pathways only.

#### Routine-to-conditional decision pathway (abbreviated)

**Domain 1 (CYP capacity):** If CYP protein abundance is below age-expected or toxicology suggests impaired clearance → consider CYP IHC (context), PGx panel (if not already performed), and (rarely) activity assays only if viability/QC criteria are met.

**Domain 2 (Cytokine load):** If IL-6/CRP are elevated or infection workup suggests inflammation → consider expanded cytokine panel (contextual) and/or tissue IHC as available.

**Domain 3 (Redox balance):** If tissue F2-isoprostanes are moderate/severe or histology suggests mitochondrial injury → consider EM (only if EM-grade fixation obtained) and/or mtDNA sequencing; expanded oxidative markers only if specified and validated.

**Domain 4 (Neurochemical integrity):** If brainstem histology/IHC shows abnormalities → consider channelopathy/epilepsy gene panel; optional contextual neurochemistry/neurosteroids when frozen tissue and QC permit.

**Domain 5 (Xenobiotic/metals):** If tox/metals are abnormal or unexplained PK is suspected → consider excipient profiling, metal speciation, and repeat metals (liver/bone) as confirmatory.

### 5. Documentation and reporting (operational)

5.1 Record specimen source, matrix, PMI, processing times (collection→separation/freeze), and storage conditions for each analyte.5.2 Document which conditional assays were performed in response to routine abnormalities.5.3 Retain frozen aliquots when feasible for confirmatory or future analysis.

## Appendix D. Technical Methods, QC, and Feasibility Constraints

### Scope

This appendix specifies analytic platforms, minimum QC/validity criteria, and feasibility constraints for postmortem assessment across the five MVI domains and CMSP anchors. It is designed to prevent preanalytical artifact from being misinterpreted as biology. Scoring thresholds and worked examples are provided in Appendix E.

### General QC and validity principles (applies to all streams)

- Use matrix-appropriate reference materials and internal standards where applicable.
- Document PMI, temperature history, and processing times; interpret results as PMI-dependent when specified.
- Treat results as **NS for scoring** when specimen identity, cold chain, or processing targets are not documented or not met.
- Avoid repeat freeze-thaw; aliquot and store at -80°C when feasible.

### 1. Domain 1 — CYP450 capacity (protein abundance anchor)

#### 1.1 Primary method (preferred)

**Targeted LC-MS/MS proteomics** (MRM/SRM or PRM) on liver homogenate/microsomal fraction, using stable-isotope labeled internal standards for isoform-specific peptides. Output as absolute abundance (e.g., pmol/mg microsomal protein) and/or normalized to **% age-matched expected** for Domain 1 scoring (Appendix E).

#### 1.2 Acceptable alternative methods

- **Western blot** (isoform-specific antibodies; densitometry normalized to total protein) where LC-MS/MS is unavailable.
- **CYP IHC (FFPE liver)** as contextual support (zonation/pattern), not a preferred quantitative anchor.

#### 1.3 Conditional methods (feasibility-dependent)

- **CYP mRNA (RT-qPCR/RNA-seq):** only when tissue is snap-frozen promptly and RNA QC (e.g., RIN/fragment analysis) passes.
- **CYP catalytic activity assays:** viability/QC-qualified only (very short PMI, verified cold chain, rapid recovery, internal QC acceptance). Not recommended as a routine postmortem anchor.

#### 1.4 Pharmacogenetic genotyping (contextual/etiologic)

PCR+Sanger or NGS PGx panel is feasible postmortem using blood or tissue DNA. For CYP2D6, include CNV/structural variant calling (e.g., qPCR/MLPA or validated NGS CNV pipeline) to avoid misclassification.

### 2. Domain 2 — Cytokine load (IL-6 anchor; CRP complementary)

#### 2.1 IL-6

Immunoassay (ELISA/chemiluminescent/bead-based) on femoral serum (preferred) or EDTA plasma. Report pg/mL and apply the ordinal bins defined in Appendix E. Avoid cardiac blood for scoring where possible.

#### 2.2 CRP

Standard CRP (not hs-CRP), nephelometry/turbidimetry or equivalent routine platform; serum preferred; plasma acceptable per lab validation.

#### 2.3 Expanded cytokine panels (conditional/contextual)

Multiplex cytokine panels may be performed as contextual data but are more sensitive to hemolysis, delays, and freeze-thaw. TNF-α/IL-1β, if obtained, are contextual only and not required for domain scoring.

#### 2.4 Infection testing

Culture and targeted PCR panels as indicated; interpret with standard forensic microbiology practices and contamination controls.

### 3. Domain 3 — Redox status (tissue F2-isoprostanes anchor)

#### 3.1 Primary method (preferred)

8-iso-PGF2α (F2-isoprostanes) in tissue by LC-MS/MS (preferred) or GC-MS/MS. Report ng/g tissue wet weight (or explicitly dry weight if used) and score relative to matrix- and method-specific reference distributions (Appendix E).

#### 3.2 Tissue selection and corroboration

Liver is default; kidney cortex may be used as corroboration when liver is compromised or unavailable. Maintain standardized anatomic sampling to reduce variability.

#### 3.3 Conditional mitochondrial investigations

- **Electron microscopy (EM):** feasible only when EM-grade fixation (e.g., glutaraldehyde; small tissue cubes) is performed promptly; otherwise yield is low.
- **mtDNA sequencing:** feasible on postmortem DNA; interpret in genetic counseling/variant classification frameworks.

#### 3.4 Contextual physiologic amplifier (vitreous)

Vitreous glucose and β-hydroxybutyrate by routine clinical chemistry analyzers validated for vitreous or by adapted validated protocols; report mmol/L and use ordinal bins (Appendix E). These results are contextual and do not alter CMSP core.

### 4. Domain 4 — Neurochemical integrity (operationally robust anchors)

#### 4.1 Routine neuropathology anchors (preferred for operational scoring)

- **Brainstem histology (H&E)** including medullary raphe sampling.
- **Serotonergic IHC** (SERT and TPH2 preferred; 5-HT IHC acceptable where validated). Use standardized protocols and document region sampling.

#### 4.2 Conditional genetic testing (etiologic)

Channelopathy/epilepsy gene panels are feasible postmortem (DNA-based) and are not limited by rapid tissue deterioration; interpret in the appropriate clinical genetics framework.

#### 4.3 Optional contextual neurochemistry (feasibility-dependent; non-scoring)

- **5-HT and 5-HIAA** quantification (LC-MS/MS or HPLC-ECD) on *frozen* brain tissue; report values and/or ratio contextually when sampling and QC permit.

**Not recommended for routine postmortem workflows:** receptor binding assays (preservation-sensitive; research-only).

### 5. Domain 5 — Xenobiotic and metal burden

#### 5.1 Comprehensive toxicology

Perform standard screening and confirmatory testing per jurisdictional SOP. Where exposure history exists or PK is unusual, include quantitative confirmation and parent:metabolite relationships to contextualize impaired clearance.

#### 5.2 Excipient/xenobiotic profiling (conditional)

Targeted profiling for formulation compounds or specific xenobiotics when indicated by history or tox patterns; LC-MS/MS methods as available.

#### 5.3 Metals

**ICP-MS** on liver (preferred) and other matrices as indicated.

**Speciation** and **repeat tissues (liver/bone)** are confirmatory and conditional, used when initial results are elevated or borderline and interpretation requires increased specificity.

#### 5.4 Aldehydes/carbonyls (conditional; method-dependent)

DNPH derivatization with chromatographic detection (LC-MS/MS preferred for complex tissue matrices; HPLC-UV acceptable where validated) may be used for free carbonyls as a conditional xenobiotic/oxidative-stress adjunct. Clearly distinguish “free aldehydes” from “protein carbonyls” assays in reporting.

### 6. Feasibility summary (what is realistic in infant postmortem operations)

**Routine-realistic anchors:** CYP protein abundance (liver), IL-6 + CRP (femoral blood), tissue F2-isoprostanes (liver), brainstem H&E + serotonergic IHC, comprehensive toxicology + ICP-MS metals.

**Conditional/feasibility-dependent:** CYP mRNA, CYP activity assays, EM, optional frozen-tissue neurochemistry (e.g., 5-HT/5-HIAA) when prompt freezing and QC permit.

**Research-only / generally impractical:** high-resolution respirometry (O2k) and receptor binding assays.

### 7. Rationale

Appendix D consolidates analytic platform options, minimum QC/validity criteria, and feasibility constraints to reduce the risk that pre-analytic artifact or postmortem degradation is misinterpreted as biology. By separating assay execution and QC from scoring (Appendix E) and specimen validity rules (Appendix C), the framework maintains clear boundaries between measurement, eligibility, and interpretation. Methods listed as “conditional” or “research-only” are included to clarify what may be technically possible versus what is operationally defensible in routine infant postmortem settings. Results that do not meet documentation, cold-chain, or processing requirements should be reported descriptively but treated as NS for scoring purposes.

## Appendix E. Metabolic Vulnerability Index (MVI): Definition and Scoring Framework

This appendix defines the Metabolic Vulnerability Index (MVI), a standardized five-domain framework for integrating enzymatic, immune, redox, neurochemical, and xenobiotic findings in unexplained infant death investigations. The MVI supports structured, mechanistically grounded interpretation within established forensic criteria. It does not assign cause of death or establish lethality. Operational workflows and specimen validity requirements are defined in Appendix C; analytic methods and QC conventions are defined in Appendix D. Design rationale and limitations are in Appendix I. Reference archetype lookup tables and referral rows are provided in Appendix H; Domain 1 age-matched CYP reference fractions are provided in Appendix F.

### 1. Conceptual integration of domains for MVI scoring (non-procedural)

The MVI integrates results across five domains to characterize physiologic vulnerability patterns. Each domain has a limited set of required scoring anchors used to assign an ordinal score (0-3). Additional measures may be reported descriptively but do not contribute points unless explicitly defined in this appendix.

**Clarification:** Formal MVI scoring requires evaluable results across all five domains (scores may be zero). If a required anchor is **NS (not scorable)** under Appendix C validity rules, the MVI should not be computed; instead report “data-limited evaluation” with domain-level descriptive results.

### 2. Metabolic Vulnerability Index (MVI): domain scoring (0-3)

Each domain is scored 0-3 using the required anchors below. Appendix C defines “scorable vs NS” eligibility (matrix, PMI guidance window, and processing/cold-chain documentation). Appendix D defines assay platforms and QC conventions.

#### 2.1 Domain 1 — CYP450 capacity

Domain 1 is implemented as a panel-based assessment: all four CYP isoforms (CYP2D6, CYP2C19, CYP3A4, and CYP3A5) are assayed when feasible, and each isoform is recorded as scorable or NS with QC reasons. Domain 1 interpretation requires at least one scorable isoform; when multiple isoforms are scorable, the Domain 1 score is assigned using the most severe tier among clinically relevant isoforms.

**Scoring anchor uses Appendix F for MVI Domain 1:** hepatic CYP capacity is preferably based on protein abundance measurement for each isoform expressed as % of age-matched expected after appropriate normalization (Appendix D; Appendix F). CYP activity assays may be reported when QC-qualified. If only one isoform is scorable, Domain 1 is still computable, but should be transparently labeled as data-limited Domain 1.

**Interpretive guardrail (infant ontogeny):** In early infancy, absolute CYP-mediated clearance is often dominated by ontogeny and may reduce practical separation between genotype-predicted phenotype categories for many CYPs. Accordingly, Domain 1 is scored using age-binned developmental expectations (Appendix F) and interpreted in context of state-dependent modifiers (e.g., inflammation/phenoconversion per Domains 2-3), rather than as a direct translation of adult pharmacogenetic phenotype categories.

**Domain 1 rule:** When multiple isoforms are evaluated, the Domain 1 score** is** the most severe (highest) score among clinically relevant isoforms in the panel (per Appendix D).

#### 2.2 Domain 2 — Cytokine load

**Scoring anchors:** IL-6 and CRP (femoral serum preferred; EDTA plasma acceptable per Appendix C/D). These anchors are selected to be operationally feasible and interpretable within typical medicolegal workflows.

**Score bands (Domain 2 anchors):** use the CMSP scoring bins in Section 4.1 (CYP, IL-6, and CRP ordinal bins).

**Domain 2 rule (aggregation):**Domain 2 score = **max(IL-6 score, CRP score)** using scorable values

#### 2.3 Domain 3 — Redox balance

**Scoring anchor: tissue F2-isoprostanes** (8-iso-PGF2α; liver default; kidney optional corroboration), reported relative to a method- and matrix-specific reference distribution. F2-isoprostanes should be reported as ng/g wet tissue weight; for scoring, values are converted to '× reference' using a matrix- and method-specific reference distribution (Appendix D)

**Score bands (Domain 3): 0:** ≤1× ref.; **1:** 1-2× ref.; **2:** 2-4× ref.; **3:** >4× ref.

**Domain 3 rule:** If both liver and kidney are available and scorable, use the higher (more severe) tier as the Domain 3 score, and report both matrices descriptively.

#### 2.4 Domain 4 — Neurochemical integrity (modifier domain)

Domain 4 captures vulnerability signals in autonomic/arousal circuitry using operationally robust postmortem measures.

**Scoring anchors (operational):** Brainstem histology (H&E) of medulla (± pons) with adequate sampling of relevant nuclei; and/or Serotonergic IHC (SERT and TPH2 preferred; 5-HT IHC acceptable where validated).

Optional Frozen-tissue neurochemistry (e.g., 5-HT, 5-HIAA, ratios) may be reported descriptively when available, but is not required for Domain 4 scoring.

**Score bands (Domain 4):**

- **0:** no meaningful abnormality in the Domain 4 anchors
- **1:** mild abnormality (limited/patchy change or borderline shift)
- **2:** moderate abnormality (clear, consistent abnormality in one anchor set)
- **3:** severe abnormality (marked abnormality and/or convergent abnormalities across anchors)

**Domain 4 rule (aggregation):** If more than one Domain 4 anchor is available and scorable, Domain 4 score = max(Brainstem histology tier**,** IHC tier**)**, with the specific basis stated in the report.

#### 2.5 Domain 5 — Xenobiotic/metal burden (modifier/exposure context)

Domain 5 captures exogenous burden and exposure context as a modifier domain that may amplify vulnerability in the presence of constrained reserve (Domains 1-3).

**Scoring anchors (Domain 5):**

- Comprehensive toxicology (screen + confirmatory testing as indicated), and/or
- Metals screen (ICP-MS) when performed (liver preferred; other matrices as indicated).

**Score bands (Domain 5): 0:** ≤1× reference / no relevant elevation; **1:** >1× to 2× reference; **2:** >2× to <3× reference; **3:** ≥3× reference

**Domain 5 rule:** When multiple xenobiotics/metals are present, Domain 5 is scored by the highest biologically relevant fold elevation above an appropriate reference background, with confirmatory testing (speciation/repeat tissues) used when needed (Appendix D).

### 3. MVI computation, safeguards, and interpretation bands

#### 3.1 Eligibility and missingness

- Compute the MVI only when all core domains (Domains 1-3) are scorable; if any core domain is NS or unscorable, do not compute a total MVI and label the case “data-limited evaluation (core domains incomplete).
- Modifier domains (Domains 4 and 5) are scored when data are available; absence of one or both modifier domains does not preclude MVI computation.
- If one or both modifier domains (Domains 4 and/or Domain 5) are **NS**, compute the MVI using scorable domains and explicitly label the case “data-limited evaluation (modifier domains(s) unavailable).”

#### 3.2 Domain safeguards and logic constraints

- **Domain 1 safeguard:** apply normalization and age-matched comparison conventions (Appendix D; Appendix F).
- **Domain 2 safeguard:** Domain 2 is scored only using IL-6 and CRP ordinal bins defined in this appendix; expanded cytokine panels may be reported contextually but do not contribute points unless explicitly defined.
- **Logic constraint:** Elevated cytokine markers are not interpreted as immune-mediated metabolic suppression in isolation; interpretation emphasizes **cross-domain convergence**, particularly with Domains 1 and 3.

#### 3.3 Total score

MVI total score = Σ (Domain 1-5 scores), range **0-15**

When one or both modifier domains are unscorable, the total MVI reflects the sum of available domains and is interpreted with explicit notation of missing modifiers.

Interpretation bands: **0-4:** low; **5-8:** intermediate; **9-12:** high; **13-15:** critical

#### 3.4 Archetype rule (Appendix H lookup)

Archetypes (Ref #1-14) are interpretive reference patterns that summarize recurrent configurations across domains. They support standardized interpretation and do not function as diagnostic entities.

Lookup procedure (Appendix H):

1. Count the number of domains with score >0.

2. Use the Appendix H table for 1-, 2-, 3-, or 4/5-domain patterns to select the matching domain combination.

3. Referral rows (“— (Referral)”) route to the appropriate Ref #1-14 archetype and specify how to record remaining domains as modifiers/exposure context.

### 4. CMSP: interpretive coherence summary (non-scoring for MVI)

The Cytokine-Metabolic Suppression Profile (CMSP) is an interpretive coherence summary used to describe immune-associated metabolic suppression signals within the broader Metabolic Vulnerability Index (MVI). The CMSP is not an independent scoring system and does not modify MVI domain scores or cause-of-death certification.

**Conceptual relationship of CMSP to CYP450 capacity (Domain 1):** The CMSP summarizes the severity and coherence of immune- and redox-associated suppression signals (Domains 2-3), while CYP450 capacity (Domain 1) represents baseline metabolic reserve. Accordingly, CYP450 capacity is reported alongside the CMSP to contextualize suppression signals but is not mathematically combined with the CMSP score.

This separation preserves the distinct interpretive roles of capacity versus suppression: Domain 1 reflects how much reserve is available, whereas the CMSP reflects whether immune or oxidative processes plausibly suppress that reserve at the time of death. Reporting both together enables integrated interpretation without additive inflation or causal inference.

#### 4.1 CMSP scoring (ordinal bins 0-3)

**CMSP Core Calculation:** The CMSP Core represents the magnitude of immune-associated metabolic suppression signals and is calculated as:

**CMSP core = max(IL-6, CRP) (Range 0-3)**

The final CMSP score is calculated as:

**CMSP = max(CMSP core, F2-isoprostanes)**

**Report CYP450 score (Range 0-3; same as Domain 1 MVI score)** alongside CMSP. Do not combine CYP450 score mathematically with CMSP Core or CMSP.

This logic emphasizes the strongest convergent signal of immune-linked metabolic stress while avoiding additive inflation from correlated markers.

##### 4.1.1 Vitreous glucose/BHB contextual pattern guide (non-scoring; interpretive context only)

Interpretive use (contextual amplifier): Vitreous glucose and BHB provide a descriptive perimortem metabolic-state/timing context alongside CMSP cytokine/redox findings. Minimal deviation supports narrating CMSP Core as the primary interpretive signal with little glucose/ketone disturbance, whereas more abnormal patterns can support descriptive wording such as ketosis-dominant (catabolic/fasting/illness interval) or hyperglycemia-dominant (stress hyperglycemia; potentially influenced by intake/resuscitation when applicable), interpreted with PMI and QC/NS rules.

#### 4.2 CMSP component rules

- **CYP450 Capacity component:** same as Domain 1 MVI score.
- **Missingness rule (Cytokine component):** If only one of IL-6 or CRP is scorable, the Cytokine component equals that score; if neither is scorable, the Cytokine component is NS.
- **Missingness rule (Redox component):** If tissue F2-isoprostanes is not scorable, the Redox component is NS.
- Interpretation emphasizes **cross-**domain convergence (Domains 1-3), not isolated abnormalities.

**Reporting format:**

Cytokine component = max(IL-6, CRP)“CMSP: max(Cytokine component, F2-IsoPs) / CYP450 Capacity = Domain 1 score;

Amplifier: BHB = *x* (contextual); Glucose = *y* (contextual).”

### 5. Worked example (hypothetical case; illustrative)

This worked example illustrates structured application of the MVI framework, including normalization, domain scoring, and archetype classification. It is presented to clarify method execution rather than to make claims about case frequency.

**Infant age:** 2 months

**Postmortem interval:** ≤12 hours

**Step 1: raw laboratory values (illustrative)**

**Domain 1 (CYP activity example retained for illustration):**

- Measured activity: CYP3A4 = 14 pmol/min/mg; CYP2D6 = 11 pmol/min/mg; CYP3A5 = 16 pmol/min/mg; CYP2C19 = 18 pmol/min/mg
- Adult reference (lab-specific; Adult = 100): CYP3A4 = 400; CYP2D6 = 100; CYP3A5 = 160; CYP2C19 = 80 pmol/min/mg

**Domain 2 (cytokine anchors):**

- IL-6 = 82 pg/mL
- CRP = 6 mg/L

**Domain 3 (redox anchor):**

- Tissue F2-isoprostanes = 3× reference (illustrative)

**Domain 4 (operational anchors):**

- Brainstem: mild serotonergic IHC reduction (illustrative)

**Domain 5 (xenobiotic/metal anchors):**

- Aluminum (liver) = 6.8 ng/mg (illustrative; scoring depends on reference framework)

**Contextual (non-scoring physiologic amplifier):**

- Vitreous β-hydroxybutyrate (BHB): 3.8 mmol/L → Score 1 (contextual)
- Vitreous glucose**:** 12 mmol/L → Score 1 (contextual)

**Step 2: normalize CYP activity to Adult = 100 (illustrative)**

CYP3A4 %Adult = (14 ÷ 400) × 100 = 3.5%; CYP2D6 %Adult = (11 ÷ 100) × 100 = 11%CYP3A5 %Adult = (16 ÷ 160) × 100 = 10%; CYP2C19 %Adult = (18 ÷ 80) × 100 = 23%

**Step 3: compare to age-matched developmental fractions (Appendix F, Section 2; illustrative)**

Use age bin: 1-2 months.

Example (illustrative expectations):

- CYP3A4 expected min = 20%Adult; measured 3.5%Adult → 17.5% of expected; tier = 3 **Domain 1 score = 3 (CYP3A4 has highest tier of measured isoforms)**
- CYP2D6 expected min = 15%Adult; measured 11%Adult → 73% of expected; tier = 1
- CYP2C19 expected min = 25%Adult; measured 23%Adult → 92% of expected; tier = 0

Highest severity across evaluated isoforms (CYP3A4) → **Domain 1 score = 3**

**Step 4: score Domain 2 (IL-6 and CRP bins)**

- IL-6 = 82 pg/mL → **Score 2** (moderate)
- CRP = 6 mg/L → **Score 0** (normal)

**Domain 2 score = max(2, 0) = 2**

**Step 5: score Domain 3 (tissue F2-isoprostanes)**

- F2-isoprostanes = 3× ref → **Score 2** (moderate)

**Domain 3 score = 2**

**Step 6: score Domain 4 (brainstem anchors)**

- Mild serotonergic IHC abnormality → **Domain 4 score = 1**

**Step 7: score Domain 5 (xenobiotic/metal burden)**

Assign tier based on the reference framework applied to the detected xenobiotic/metal(s) and confirmatory context (Appendix D).(For illustration) if aluminum corresponds to a moderate tier → **Domain 5 score = 2**

**Step 8: compute the MVI**

**Total MVI = 10 / 15** (high vulnerability band)

**Step 9: compute CMSP (interpretive only)**

- CYP450 capacity component = Same as MVI CYP450 score 3 = **3**
- Cytokine component = max(IL-6 = 2, CRP = 0) = **2**
- Redox component = F2-IsoPs score 2 = 2

CMSP = max(Cytokine component score 2, Redox component score 2) = 2

CYP450 capacity = 3

Report CYP450 capacity (Domain 1 score) alongside CMSP; do not mathematically combine.

Amplifier (contextual): Vitreous glucose score = 1; vitreous BHB score = 1 → Minimal deviation pattern (Appendix E, Section 4.1.1)

**Step 10: identify archetype (Appendix H)**

Count non-zero domains (illustrative: 5) → use 4/5-domain table; select matching combination; record Domains 4 and/or 5 as modifiers per referral row.

**Step 11: interpretive summary (illustrative)**

This case demonstrates a high MVI (10/15) with convergence across core domains (Domains 1-3), consistent with constrained metabolic reserve accompanied by immune and oxidative burden. Domains 4-5 provide modifier and exposure context and are documented accordingly in archetype classification. The CMSP is reported as an internal coherence summary reflecting the severity of Domains 2-3 signals, with CYP450 capacity (Domain 1 = 3) reported alongside but not incorporated into CMSP calculation. Vitreous glucose and β-hydroxybutyrate show a Minimal deviation contextual pattern (Appendix E, Section 4.1.1), providing limited perimortem metabolic-state context without modifying CMSP Core or any MVI outputs.

### 6. Cases outside the detection envelope of the MVI

Although the MVI provides a structured approach to identifying vulnerability patterns, some infant deaths can be lethal with minimal measurable disturbance across the five domains. In such cases, MVI scores may be low or normal despite lethality; this reflects a detection limitation rather than absence of mechanism. When MVI scores are low or minimal, additional etiologic investigations may still be indicated based on case context (e.g., targeted genetic evaluation for arrhythmia/channelopathy or epilepsy syndromes); these assessments are interpreted within standard forensic and clinical-genetics frameworks and are not part of MVI scoring.

**Interpretive note:** The MVI is interpreted only within a complete forensic evaluation and does not substitute for structural, traumatic, cardiopulmonary, infectious, or neuropathologic assessment.

### 7. Integrated report template (optional)

**Case ID:** ________ **Date/Investigator:** ____________________

A. Demographics/Circumstances: age; gestational age; weight; illness; medications/exposures; environment/sleep context.

B. Genetics (if performed): CYP2D6/CYP2C19/CYP3A4/5 genotype.

C. Domain 1 (CYP capacity): capacity values; normalization; age-matched comparison; score.

D. Domains 2-3 (immune/redox): IL-6 and CRP bins (Domain 2 score); tissue F2-IsoPs tier (Domain 3 score); infection workup summary; vitreous amplifier if measured.

E. Domain 4 (neurochemical integrity): brainstem H&E and serotonergic IHC summary; score; specimen limitations if any.

F. Domain 5 (xenobiotic/metal burden): tox summary; metals summary; confirmatory testing where applicable; score.

G. Summary outputs: Domain 1-5 scores; Total MVI; Archetype (Ref #); CMSP (if computed; interpretive only).

H. Interpretive integration: one paragraph describing what drove the score, whether Domains 1-3 converge, and how modifier domains contextualize vulnerability; include “does not establish cause of death” safeguard if not stated elsewhere.

### 8. Rationale (brief)

The MVI provides a reproducible, ordinal method to integrate metabolic reserve (Domain 1), immune load (Domain 2), oxidative burden (Domain 3), neurochemical integrity (Domain 4), and xenobiotic/metal burden (Domain 5) to support mechanistic contextualization of vulnerability patterns in unexplained infant deaths alongside standard forensic practice. CMSP provides an internal coherence summary of immune-associated metabolic suppression patterns using operationally feasible markers and does not modify MVI scoring, archetype assignment, or certification.

## Appendix F. CYP Protein Abundance and Activity Reference Tables

Tables 1 and 2 provide adult plausibility bounds to support initial assay validation and confirm that each laboratory's adult reference material is within a physiologically reasonable order of magnitude for each isoform. These plausibility bounds are not used for scoring.

For interpretation of infant cases, CYP absolute protein abundance is the primary measurement because quantified apoprotein remains measurable and comparatively stable over longer postmortem intervals, whereas CYP catalytic activity degrades rapidly and is more sensitive to pre-analytical conditions. Laboratories using different assay configurations (e.g., probe substrates, substrate concentrations, incubation conditions, microsomal preparation, analytic platform) should not expect to reproduce the absolute values shown here; instead, results should be interpreted on a normalized (%Adult) scale.

Each laboratory establishes an internal adult normalization anchor by assaying pooled adult human liver microsomes under validated conditions. This measured adult value defines Adult = 100% for that laboratory and isoform. Infant CYP measurements are then expressed as:

%*Adult* = (*Infant value* ÷ *Adult reference*) × 100

Normalized infant values are interpreted using the age-matched Master Developmental Reference Tables (1.1-1.4 for protein abundance; 2.1-2.4 for activity) corresponding to the infant's age. Isoforms are categorized as 0 (normal/minimum expected), 1 (mildly reduced), 2 (moderately reduced), or 3 (severely suppressed**)** based on fixed proportional reductions below the age-specific minimum. Numeric MVI/CMSP scoring criteria and worked examples are defined in Appendix E.

**Genotype/phenotype context:** When genotype predicts physiologically minimal enzyme function (e.g., CYP3A5 non-expressers), low measured protein/activity is considered normal for that isoform and suppression grading is not applied (see isoform-specific notes). Otherwise, apply applicable grading using Section 1 for CYP protein abundance tables or Section 2 for CYP activity tables, including when genotype is unavailable or indeterminate.

### 1. Adult hepatic CYP Protein Abundance Reference Values (Interpretive Context)

Adult hepatic CYP protein abundance (HLM) — plausibility bounds (not for scoring; for assay validation / order-of-magnitude checks)

#### Rationale: Why CYP protein abundance can proxy “CYP450 capacity” in postmortem infant liver

**Conceptual definition used in Appendix H.** In this framework, CYP450 capacity refers to the liver's *structural metabolic reserve* for a given isoform—i.e., the amount of enzyme available to support intrinsic clearance under physiologic conditions. CYP protein abundance (pmol CYP per mg microsomal protein) is a direct measure of enzyme amount and therefore serves as a practical proxy for this reserve when interpreted on a normalized (%Adult) scale and within age-matched developmental expectations.

- Protein abundance represents enzyme “amount,” a core determinant of capacity

For any CYP-mediated pathway, observed metabolic rate can be conceptualized as:

Activity is proportional to (Enzyme amount) × (Catalytic competence) × (Cofactors/redox) × (Inhibition/activation)

Protein abundance captures the enzyme amount term. Because developmental ontogeny, many genotype-linked differences (e.g., expressor vs non-expressor states), and chronic regulatory changes primarily manifest through altered enzyme amount, protein abundance provides a stable and interpretable measure of isoform-specific metabolic reserve.

- Protein is typically more postmortem-robust than activity

In autopsy material, catalytic activity degrades rapidly due to loss of cofactors, membrane integrity, and redox function, as well as proteolysis and pre-analytical variation (PMI, temperature, handling). In contrast, quantified CYP apoprotein can remain measurable for longer postmortem intervals, even when probe-substrate activity is markedly reduced or absent. For this reason, protein abundance is treated as the primary measurement for Domain 1 “CYP capacity,” while activity is treated as a secondary, conditional readout used when QC-qualified.

- Adult normalization makes protein tables portable across laboratories

Absolute CYP protein values vary across laboratories due to differences in microsome preparation, digestion efficiency, peptide selection, calibration standards, and instrument response. Normalizing each isoform to a laboratory's own pooled adult reference (Adult = 100%) converts results to a common physiologic scale (%Adult). This enables consistent comparison of infant samples against age-matched minimum expectations even when absolute numbers differ across sites.

- Why activity remains secondary rather than eliminated

Activity assays are the most direct measure of functional intrinsic clearance, because they incorporate:

- enzyme amount,
- catalytic competence,
- cofactors/redox status, and
- inhibition/activation.

However, those same dependencies make activity more fragile postmortem and more sensitive to assay configuration (probe choice, substrate concentration, incubation conditions). Accordingly, activity is retained as a secondary confirmatory measurement: it can strengthen inference when concordant with protein, and it can flag **acute** functional suppression (e.g., inhibition or cofactor failure) when activity is disproportionately low relative to protein.

- Genotype/phenotype context prevents misclassification (especially CYP3A5)

Certain isoforms have physiologically low/absent function in normal individuals based on genotype/phenotype (e.g., CYP3A5 non-expressers; CYP2D6 poor metabolizers). In these cases, low protein or low activity is **not suppressive**. Therefore:

- adult reference anchors should exclude poor-metabolizer phenotypes unless genotype-stratified references are intentionally used, and
- isoform suppression grading is applied only when the genotype/phenotype indicates the enzyme is expected to be functionally present (e.g., CYP3A5 suppression grading applies only to *1 carriers).

Protein abundance estimates structural metabolic reserve; activity provides conditional evidence of functional competence and acute inhibition. Both are interpreted after adult normalization and age-matched comparison; scoring procedures are defined in Appendix E.

### 2. Adult hepatic CYP Activity Reference Values (Interpretive Context)

#### Rationale: Why CYP activity can proxy “CYP450 capacity” in postmortem infant liver (secondary/conditional)

**Conceptual role in Appendix H:** CYP catalytic activity (probe-substrate turnover per mg microsomal protein) provides a functional proxy for CYP450 capacity because it reflects the combined effect of enzyme amount and catalytic competence under assay conditions. When quality-controlled, activity can therefore serve as a practical readout of isoform-specific metabolic competence and intrinsic clearance potential.

- **Activity is a direct functional readout—when QC-qualified**

Activity assays incorporate multiple determinants of in situ metabolic performance, including:

- enzyme amount,
- catalytic competence (active vs inactive apoprotein),
- availability of cofactors/redox partners, and
- inhibition/activation by endogenous or exogenous compounds.

For this reason, when a postmortem microsomal preparation is adequately preserved and assay conditions are validated, **activity can approximate functional metabolic capacity** more directly than protein abundance alone.

- **Why activity is treated as secondary in postmortem material**

In autopsy tissue, measured activity is more vulnerable than protein to:

- postmortem interval and temperature effects,
- loss of cofactors/redox function,
- membrane integrity changes,
- pre-analytical handling variability, and
- assay configuration differences (probe substrate, concentration, incubation conditions).

Accordingly, activity is used as a secondary/conditional proxy in this appendix: it strengthens inference when concordant with protein abundance and can reveal acute functional impairment (e.g., inhibition or redox failure) when activity is disproportionately low relative to protein.

**Genotype/phenotype context remains essential**

Genotype-driven physiologic low-function states (e.g., CYP3A5 non-expressers; CYP2D6 poor metabolizers) can yield minimal activity that is **normal** rather than suppressive. Therefore, activity-based suppression grading should be applied only when the enzyme is expected to be functionally present (see isoform-specific notes).

**Practical interpretation statement:** In this framework, protein abundance estimates structural metabolic reserve, while activity provides conditional evidence of functional competence and acute inhibition; both are interpreted only after adult normalization and age-matched comparison, with scoring procedures defined in Appendix E.

## Appendix G. Operational Roles and Responsibilities (Appendices C-E)

This appendix maps the operational tasks in Appendices C-E to the professional roles responsible for specimen handling, analytic testing, quality control, and reporting in unexplained infant death investigations. It is implementation-focused and does not modify analytic thresholds, scoring rules, or cause-of-death certification.

### Operational Constraints and Safeguards

- CYP450 activity assays should be performed only by laboratories with validated CYP activity methods (probe selection, linearity, QC, and defensible Adult=100 normalization references).
- Electron microscopy should be ordered only when redox markers and/or histology suggest mitochondrial injury.
- Pharmacogenetics must be interpreted with enzyme activity and (when performed) IHC to confirm genotype-phenotype concordance.

### Rationale

Most medicolegal investigations rely on routine autopsy methods that may not detect metabolically mediated vulnerability. This appendix provides an operational map linking roles to the workflows in Appendices C-E to support consistent specimen handling, testing, QC, and reporting.

## Appendix H. Reference Archetypes for MVI Pattern Interpretation

This appendix provides standardized reference archetypes (Ref. #1-14) for interpreting MVI domain-pattern configurations.

**Lookup rule (archetype selection):** Identify which domains have non-zero scores and count how many. Use the table corresponding to 1-, 2-, 3-, or 4/5-domain patterns and select the row that matches the domain combination present. Rows labeled “— (Referral)” direct the reader to an existing archetype (Ref. #1-14) to avoid creating overlapping or duplicate archetype descriptions; any additional non-zero domains are documented as modifiers/exposure context as specified in the referral row.

## Appendix I. Design Rationale & Interpretive Safeguards of the MVI

The design principles, scope, and interpretive safeguards of the MVI are summarized below to address common methodological and interpretive questions.
